# Zwitterion-doped liquid crystal speckle reducers for immersive displays and vectorial imaging

**DOI:** 10.1038/s41377-023-01265-5

**Published:** 2023-09-22

**Authors:** Yihan Jin, Nathan P. Spiller, Chao He, Grahame Faulkner, Martin J. Booth, Steve J. Elston, Stephen M. Morris

**Affiliations:** Department of Engineering Science, University of Oxford, Parks Road, Oxford, OX1 3PJ UK

**Keywords:** Liquid crystals, Imaging and sensing, Optoelectronic devices and components, Displays

## Abstract

Lasers possess many attractive features (e.g., high brightness, narrow linewidth, well-defined polarization) that make them the ideal illumination source for many different scientific and technological endeavors relating to imaging and the display of high-resolution information. However, their high-level of coherence can result in the formation of noise, referred to as speckle, that can corrupt and degrade images. Here, we demonstrate a new electro-optic technology for combatting laser speckle using a chiral nematic liquid crystal (LC) dispersed with zwitterionic dopants. Results are presented that demonstrate when driven at the optimum electric field conditions, the speckle noise can be reduced by >90% resulting in speckle contrast (*C*) values of *C* = 0.07, which is approaching that required to be imperceptible to the human eye. This LC technology is then showcased in an array of different display and imaging applications, including a demonstration of speckle reduction in modern vectorial laser-based imaging.

## Graphical abstract

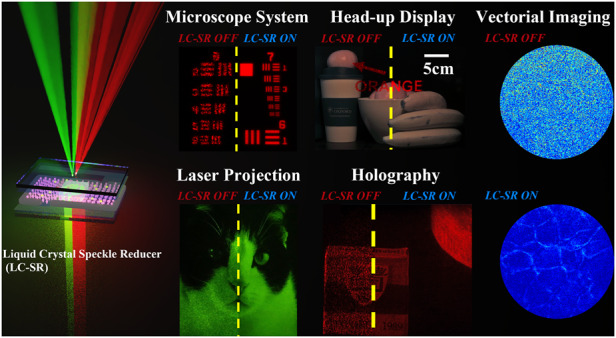

## Introduction

Laser light sources are advantageous for imaging and display applications as they can combine high brightness emission with low beam divergence and narrow spectral linewidth^1–5^. These sources of illumination can also be readily combined with optical waveguides and optical fibers of different geometries and configurations. Additionally, lasers are highly desirable, if not essential, when it comes to applications such as optical coherence microscopy, near-eye displays, holography, laser projectors, optical microscopy for biomedical applications, and polarization sensing. In the latter case, polarization sensing plays an important role in areas such as quantified polarization entropy^6^, quantum physics, in the form of spin-orbital conservation^7,8^, and material characterization and clinical diagnosis, such as label-free cancer detection^9–11^.

For many of these applications, speckle is an unwanted artifact that degrades the quality of the imaging system and, in some cases, prevents the advantageous features of laser sources from being fully exploited^12–15^. This phenomenon of speckle occurs when a highly coherent, narrow linewidth light source passes through a highly scattering material or is reflected by an optically rough surface; for example, in the form of highly scattering optical elements or biomedical tissue samples, respectively. Each facet on the scattering surface reflects the incident light and generates a randomly phased element, which contributes to an intensity fluctuating interference pattern in the image plane. This leads to either constructive or deconstructive interference which gives rise to the appearance of a granular intensity pattern when observed by the human eye, for example. Speckle has not been limited to the visual spectrum but has also been observed with X-ray sources^16,17^, medical ultrasound^18,19^, radar^20^ and other applications that use coherent radiation.

The degree of speckle can be quantified by the speckle contrast parameter, *C*, that was originally introduced by Goodman^21–24^. This speckle contrast parameter, which requires that the image is of a uniform average intensity, can be defined as

1 $$C=\frac{{\sigma }_{I}}{\bar{I}}$$

where *σ*_*I*_ is the standard deviation of the intensity values in the image plane of interest and $\bar{I}$ is the average intensity value. The lower the speckle contrast value *C*, the more uniform the intensity across the image. For fully developed speckle, when the most severe speckle pattern is present, the speckle contrast parameter *C* and the signal to noise ratio *S*/*N* (the inverse of the speckle contrast parameter) are both equal to 1. From the perspective of perception, research has shown that a speckle pattern becomes imperceivable to the human eye when *C* is lower than a value of 0.04^25^.

In the past, a range of different methods have been developed to minimize the presence of speckle. These can be loosely divided into four separate categories: polarization decorrelation^12^, angular decorrelation^26–28^, wavelength decorrelation^29^, and spatial decorrelation^2^. All these methods aim to reduce the spatial coherence and/or temporal coherence of the laser light. Each method can reduce the speckle to a certain degree instantaneously or over a fixed period of time. With an active speckle reducing element placed in the propagation path of a light source, the speckle contrast takes the form^12^

2 $$C=\frac{\sqrt{{\sum }_{n=1}^{N}{\bar{{I}_{n}}}^{2}}}{{\sum }_{n=1}^{N}\bar{{I}_{n}}}$$

where *N* is the number of decorrelated speckle patterns. Assuming that all of the speckle patterns have the same average intensity value, then the speckle contrast can be reduced by $\frac{1}{\sqrt{N}}$.

Generating decorrelated speckle patterns can be realized by using a range of different techniques such as the introduction of a depolarizing screen^12^, the use of an array of lasers aligned at different angles relative to the receiver^30^, an array of lasers emitting at different wavelengths, the use of a low spatial coherence light sources such as a random laser^2^, or the use of a rotating ground glass diffuser^31^. In recent reports, approaches taken to improving imaging quality can be classified as involving either the introduction of optical elements to optimize the laser source or to employ numerical methods, such as neural networks^32^, to reconstruct the data that is recorded. The former has the advantage that it fundamentally improves the *S/N* ratio of the optical system.

With regards to the introduction of a physical optical element, the most common approach is to place a rotating ground glass diffuser (RGGD) before the collection optics in a wide field of view imaging system^31^. Previous research has shown that the speckle can be decreased to an acceptable level if the rotation speed of the RGGD (roughness average = 1.57 µm) is >2π rad/s^33^. In this previous study, the speckle reducer was placed in a laser projection system with a diode-pumped solid-state laser, mimicking a head-up display (HUD) system, where it was demonstrated that a reduction of the speckle contrast by ≈95% could be achieved. While this approach can mitigate the appearance of speckle, it comes at the expense of some considerable drawbacks such as the presence of mechanical vibrations caused by the gear and the stepper motor, which is not desirable for measurements/experiments requiring a high degree of sensitivity or where low mass and volume are key drivers.

Another approach that involves the introduction of an optical element is the use of a mechanically rotating ball lens in a laser projection system^34^, where it was shown that the speckle contrast could be reduced by 54%. However, in this case the motor driven ball lens consisted of a rotating shaft that deviates from the center of the ball lens leading to a series of separate spots while the lens is rotated. To obtain a stable light spot on the projection screen while the ball lens was rotating, the speed of rotation must be very fast, with a rotation speed of 5000 rotations per minute^34^. Additionally, the aberration caused by the deliberate deviation distance from the ball center to the rotation axis would be a problem when the projection area is large. Again, even though this approach is effective at reducing speckle it comes at the price of mechanically moving components.

Except for conventional optical elements, a phase mask diffuser encoded with a 64^th^ order of Hadamard matrix pattern (*H(64)*)^13,35^ has been employed to generate cyclic random phase masks corresponding to the Hadamard orthogonal functions. This method has been shown to reduce the speckle contrast by $\frac{1}{\sqrt{64}}$ when the phase mask was placed at the intermediate image plan and set in transverse oscillatory motion by a voice coil, combining with scanning action of the Grating Light Valve (GLV)^36,37^ laser projection system^13,35,38^. The reduction in speckle in this case is very impressive, but the sheer cost, induced vibration and size of the GLV system makes these impractical for many applications.

An alternative approach is to employ electro-optic materials that can be compact in size, free from moving parts, and that can generate a series of statistically independent speckle patterns within a finite period of time when a voltage is applied to the material. Towards this end, liquid crystalline materials have been shown to be promising candidates^14,15,39–44^. The combination of the birefringence and the reorientation of the director when an external electric field is applied makes it particularly appealing for the generation of random phase or polarization states that can lead to decorrelated speckle patterns. Research has been carried out previously to demonstrate the potential of liquid crystal (LC) devices for speckle reduction. One example is the work by Zhaomin Tong et al. who demonstrated a 55% reduction by using a polymer dispersed nematic liquid crystal (PDLC) diffuser^42^. Separately, Hayato Ishikawa et al. have shown that ferroelectric LCs (FLC) dispersed with SiO_2_ particles can reduce the speckle by >40% when voltages of the order of 150 V were used^43^. A significant reduction was demonstrated by Andreev et al. who generated a random refractive index profile across the FLC resulting in a 50% reduction in speckle^44^. These findings were also supported in studies conducted by Furue et al. where it was shown that the speckle could be reduced using polymerized FLCs or surface stabilized FLCs as well as using photocurable monomer dispersed nematic LCs^14,15^.

In recent work, we have proposed a strategy of combining chiral nematic LCs with either ionic^41^ or redox^39^ dopants to generate electrohydrodynamic instabilities (EHDI) that can reduce the speckle contrast to values of *C* = 0.22 ± 0.02 or *C* = 0.11 ± 0.02, respectively. In this previous work, the application of a low-frequency AC electric field to the LC device results in competing torques between the conductivity and the dielectric coupling. This creates a dynamic scattering state for light that can be used to generate a series of time-sequential angular decorrelated speckle patterns that average out over the integration time of the detector (e.g., the human observer), which in turn leads to a significant reduction in the speckle contrast.

In the present study, we demonstrate a new configuration that involves the dispersion of zwitterions (in the form of Reichardt’s dye) into a chiral nematic LC host and show how this device can reduce the speckle contrast in a range of different display and imaging applications. Zwitterions have been investigated in different applications previously including in luminescent perovskite nanocrystals^45^ and as the supplier of charges in self-targeting nanocarriers for clinical applications^46^. In the context of LC devices, recent research has demonstrated a promising new smart window technology by doping a negative dielectric anisotropy LC with zwitterions^47^. For our work, we also selected Reichardt’s dye, which is an organic dye that is known to be zwitterionic. Commonly used electrolytes such as ionic dopants and redox dopants typically suffer from electron accumulation at the surfaces resulting in device degradation after repeated usage because of the imbalance of the anionic and cationic composition. Zwitterions, with their equal amounts of negatively and positively charged functional groups in one molecule, are considered as a great alternative for overcoming these issues. Here we show that these zwitterionic-based LCs have great potential for speckle reduction and that the speckle contrast can be reduced to a level that is imperceptible to the human eye while maintaining a device transmission of around 50%. This new speckle reducer is then showcased in six different application scenarios which includes a wide-field microscope, holography, HUD, high-intensity laser projector, and a Mueller Matrix microscope with transmissive and back-reflection geometries.

## Results

### Speckle reduction using a zwitterionic-doped LC reducer

The LC speckle reducers (LC-SR) consisted of a short-pitch positive dielectric anisotropy chiral nematic mixture dispersed with different concentrations of a zwitterionic dopant (Reichardt’s dye) that were capillary filled into glass cells that consisted of transparent conductors coated onto the inner surfaces of the glass substrates to facilitate the application of an electric field. More information about the mixtures and device fabrication processes are provided in the Materials and Methods section. Before testing these devices in different imaging and display applications, the LC-SR were first characterized in terms of the amount of speckle reduction that was achievable for different mixture compositions, LC layer thicknesses and operating temperatures. For each mixture formulation, we performed a scan of the speckle contrast for a range of electric field conditions (i.e., amplitude and frequency) in order to identify the conditions required for maximum speckle reduction. Further details about the experimental techniques employed to characterize the speckle contrast are described in the Materials and Methods section.

**Fig. 1:**
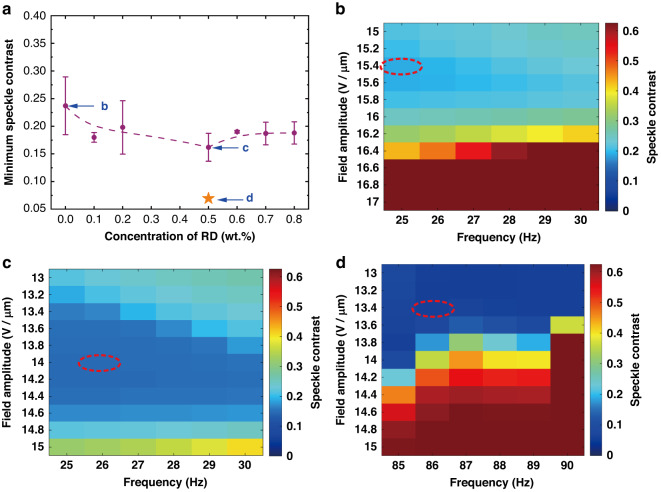
Reduction in the laser speckle contrast using a zwitterionic-doped chiral nematic liquid crystal speckle reducer (LC-SR). **a** Plot of the speckle contrast, *C*, as a function of the concentration of the zwitterionic dopant (by weight percentage) for LC-SRs that consisted of 20 μm-thick LC layer at a temperature of 25 °C (purple closed circles) and a 40 μm-thick LC layer at a temperature of 50 °C (orange star). The dashed line in the figure is simply to guide the eye and has no physical meaning. Example colormaps of the speckle contrast are shown for a range of electric field amplitudes and frequencies for: **b** the host chiral nematic LC mixture without any zwitterionic dopant; **c** with 0.5 wt.% zwitterionic dopant for a *d* = 20 μm LC layer, and **d** 0.5 wt.% zwitterionic dopant for a *d* = 40 μm LC layer at a temperature of 50 °C. These colormaps correspond to the data points and concentrations of zwitterionic dopant labeled in (**a**). The smallest speckle contrast value recorded (for a particular combination of electric field amplitude and frequency—referred to as the optimum electric field conditions) are encircled by a red dashed ellipse in each plot

As shown in Fig. 1, the maximum reduction in the observed speckle contrast was found to depend upon the concentration of the zwitterionic dopant (Reichardt’s dye) dispersed into the chiral nematic LC host (the speckle contrast for different pitch values and concentration of zwitterionic dopant is presented in Fig. S1 in the Supplementary Information). Figure 1a shows the minimum speckle contrast recorded as a function of the concentration of Reichardt’s dye. At very low concentrations of dopant (RD < 0.3 wt.%), EHDI were insufficient in most cases to generate enough light scattering required to reduce the speckle contrast to below *C* = 0.2. However, with an increase in the concentration of the zwitterionic dopant, the turbulence and dynamic scattering increased, resulting in a better reduction in the speckle contrast. The optimum concentration of the zwitterionic dopant (Reichardt’s dye) was found to occur at 0.5 wt.% at which point the speckle contrast was reduced to *C* = 0.15 ± 0.01 (for an LC layer thickness of 20 μm and a temperature of 25 °C). Increasing the concentration of Reichardt’s dye (RD) further resulted in a gradual increase in the minimum speckle contrast value, although this was still found to be lower than that observed for the base chiral nematic LC mixture on its own (mixture with RD = 0 wt.%).

The mixture that exhibited the lowest speckle contrast (BL006 + 3 wt.% R5011 + 0.5 wt.% Reichardt’s dye) was further tested in a glass cell with a larger cell gap, the results of which are shown by the orange star in Fig. 1a. The smallest value of the speckle contrast, *C* = 0.07, recorded in this study was observed using a glass cell with a gap of 40 μm at a temperature of 50 °C. This corresponded to a very large speckle reduction of 90%. Figure 1b–d show scans of the speckle contrast over a range of electric field amplitudes and frequencies for the three data points (and thus three different mixture/devices) highlighted in Fig. 1a. These scans of the electric field conditions show the speckle contrast in the form of a colormap, where red colors indicate a large speckle contrast and blue colors represent a low speckle contrast^39,40^. Each mixture/device was characterized by first a low-resolution test followed by a high-resolution scan that was concentrated around the electric field conditions relating to the largest reduction in the speckle contrast.

The red dashed ellipses in the speckle contrast colormaps in Fig. 1b, c show the peak field condition for the case of no zwitterionic dopant (Fig. 1b) and with 0.5 wt.% of zwitterionic dopant (Fig. 1c). The two devices in Fig. 1b, c consisted of an LC layer that was 20 μm-thick and operated at 25 °C. It can be seen clearly that the addition of the zwitterionic dopant resulted in a substantial reduction in the speckle contrast at lower electric field amplitudes (*C* = 0.15 for the 0.5 wt.% mixture compared with *C* = 0.3 for the mixture without zwitterionic dopants). The colormap in Fig. 1d, on the other hand, is for the 0.5 wt.% mixture but in a 40 μm-thick cell operated at 50 °C. Evidently, increasing the thickness of the LC layer as well as the operating temperature can give a significant improvement of the device performance, which is in accord with the results obtained in previous work^48^. Further discussion about the dependence of the speckle contrast on the RD concentration as well as the behavior observed as the electric field amplitudes and frequencies are varied, such as the apparent transition from regions where the speckle contrast is reduced to regions where the speckle contrast shows no obvious reduction (e.g., Fig. 1b, d), can be found in the Supplementary Information. An investigation was also conducted to determine the electric field for which both the speckle contrast and the transmission of the LC-SR depicted in Fig. 1c was found to saturate (Fig. S2).

It is important to emphasize that even though the best result in terms of reducing the speckle to *C* = 0.07 was obtained at an elevated temperature, this thicker LC layer also exhibited a very low speckle contrast of *C* = 0.075 at a temperature of 25 °C, which is an improvement on LC speckle reducers reported previously even at elevated temperature (*C* ≈ 0.09 at a temperature of 55 °C)^39^.

Like the speckle contrast, the transmission and scattering haze of the LC-SR are also an important properties. To investigate the transmission characteristics, a series of speckle patterns were captured using a monochrome CCD that imaged the speckle pattern displayed on a white screen (see Materials and Methods). Each speckle pattern was formed by placing the different LC-SR devices (containing different concentrations of zwitterionic dopant) in the path of a He-Ne laser.

**Fig. 2:**
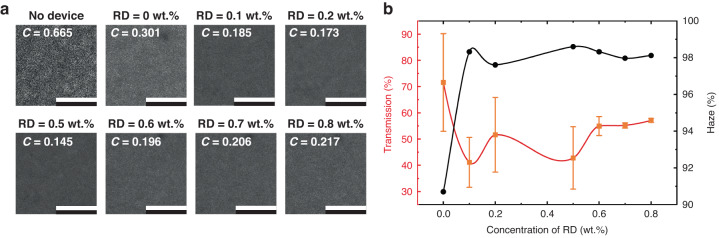
Speckle patterns and transmission recorded for zwitterionic-doped chiral nematic liquid crystal speckle reducers (LC-SRs). **a** Speckle patterns recorded by the CCD camera when the light from a He-Ne laser passed through an LC-SR for different concentrations of zwitterionic dopant. The first image shows the speckle pattern for the mixture without any zwitterionic dopant and with no electric field applied. The scale bar in the images represents a distance of 2 mm. **b** The corresponding transmission of light after passing through the LC-SR relative to the intensity recorded for no electric field applied (red solid line) and the haze value of the devices under their optimum operating conditions (the black solid line). All images and data points were captured at 25 °C and the LC layer thicknesses were *d* = 20 μm

The speckle patterns that were observed on a white screen for the different LC-SRs are presented in Fig. 2a. The top left-hand image shows the speckle pattern that was observed when the LC-SR with no zwitterionic dopant (0 wt.%) was placed in the light path and with no electric field applied, showing a very clear and obvious granular intensity speckle pattern. The subsequent images show how the speckle pattern changes with the addition of the zwitterionic dopant and when the LC-SRs were switched on. Each speckle pattern was obtained when the devices were driven at their respective optimum electric field conditions. The transmission of light after propagating through the different devices was then calculated based on the intensity recorded in the images in Fig. 2a relative to the intensity recorded in the first image when no electric field was applied to the LC-SR with no zwitterionic dopant. For each data point of transmission plot in Fig. 2b, the electric field amplitude and frequency applied to each sample corresponded to the conditions required for maximum speckle reduction. The results show that there was a decrease in the transmittance with the addition of the zwitterionic dopants, and that the lowest transmission was recorded for the sample that also exhibited the lowest speckle contrast (i.e., 0.5 wt.%).

The reduction in the transmission within these devices was mainly due to a combination of the changing macroscopic structure of the chiral nematic LCs and the efficiency of the optical arrangement at capturing the scattered light. Illustrations of the macroscopic structure and corresponding polarizing optical microscope images of the static and dynamic scattering states can be found in the Supplementary Information (Fig. S3). The macroscopic structure of the chiral nematic LCs was temporally varying while operated under different electric field conditions. This introduced different degrees of angular scattering for light propagated through the device and that was the reason why the transmission plot varies as shown in Fig. 2b. However, the distance between the end of the light pipe and the entrance pupil plane of the microscope objective (serving as a magnifier) remained constant for all experiments. Therefore, we see the transmission drops dramatically as the concentration reaches its optimum point because the diffuse angle of the light after the light pipe was so large for the best device that not all the light could be collected by the imaging microscope objective. Nevertheless, the results presented in Fig. 2b show that the relative transmittance did not drop below 45%, which is important for practical applications, and we expect the transmission values can be generally higher if the detector is placed closer to the devices or a greater collection of the devices is achieved (e.g., through the use of higher numerical aperture collection optics).

The right-hand axis in Fig. 2b depicts the corresponding scattering haze values extracted for the optimum electric field conditions (as indicated by the dashed ellipses highlighted on the examples shown in Fig. S4 in the Supplementary Information) for each concentration of zwitterionic dopant (presented as a percentage). Notably, the haze as a function of the concentration of the zwitterionic dopant exhibits an opposite trend compared to the plot of the transmission. Here it can be seen that the haze is of the order of 90% without the dopant but increases sharply to 98% with the concentration of dopant. These findings are consistent with the plots in Fig. 1b, c because the decrease in speckle contrast occurs with an increase in scattering and consequently leads to a decrease in transmission. Finally, the response time of the LC-SR device was found to be ~24 ms (Fig. S5 in the Supplementary Information), which should not be a significantly limiting factor in practical systems such as laser projectors or HUDs that are considered in more detail in the next sections.

### LC speckle reducer in a laser microscopy imaging system

**Fig. 3:**
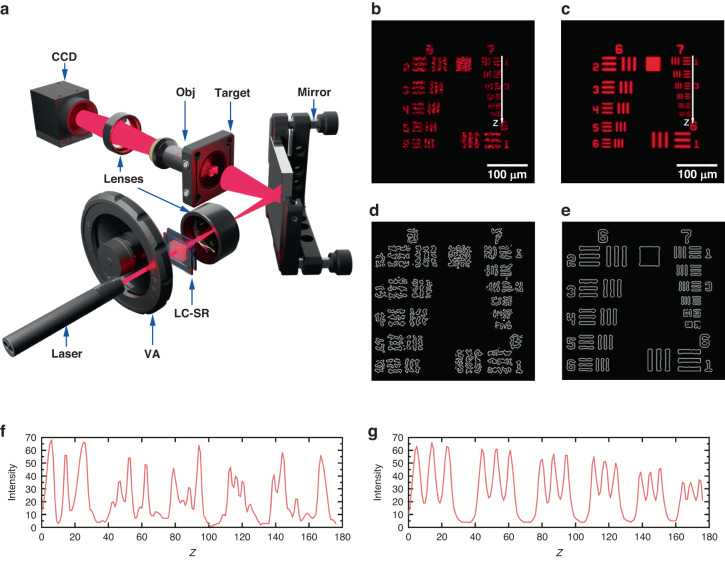
Zwitterionic-doped chiral nematic LC speckle reducer (LC-SR) in a laser microscope imaging system. **a** Schematic of the laser microscope assembly used to demonstrate the LC-SR. The best performing LC-SR was employed for this demonstration (BL006 + 3.0 wt.% R5011 + 0.5 wt.% Reichardt’s dye, *d* = 40 μm operated at a temperature of 50 °C—corresponding to a speckle contrast of *C* = 0.07). An objective (Obj) was used to collect the light from the USAF 1951 target before it was directed to the CCD camera by a focusing lens. The target was a combination of a 220-grit ground glass diffuser and a Ø1” 1951 USAF Target. **b** The image captured by the CCD camera without the LC-SR. **c** Image captured when the LC-SR was switched on. **d** The corresponding image after an edge detection algorithm (Laplacian of Gaussian) had been applied to (**b**). **e** The corresponding image when an edge detection algorithm had been applied to (**c**). **f** The intensity extracted along a line defined by *z* in (**b**). **g** The intensity extracted along a line defined by *z* in (**c**). The *z-*axis starts from group 7 element 1 to group 7 element 6

To illustrate the capability of the zwitterionic-doped chiral nematic LC-SR, we now investigate the performance of the technology in six different application scenarios. For what follows, the 40-µm-thick LC-SR consisting of the 0.5 wt.% zwitterionic dopant was used exclusively unless stated otherwise. Figure 3 demonstrates the performance of the LC-SR when it was deployed in a laser microscope imaging system. It is immediately discernible that the imaging quality significantly improved when the LC-SR was integrated into the microscope and run at the optimum electric field conditions. The compact size of the diffuser and the absence of any moving mechanical components means that it could be easily integrated into the laser microscope assembly. As can be seen by comparing Fig. 3b with Fig. 3c, the sharpness of the fringes and the visibility of the numbers adjacent to the fringes in the target being imaged were significantly improved because of the presence of the LC-SR. Using an edge detection algorithm (Laplacian of Gaussian (LoG) operator) the key features (shapes/numbers) from Fig. 3b, c were extracted. The results for alternative edge detection algorithms are presented in Figs. S6 and S7 in the Supplementary Information.

The results from the edge detection emphasize clearly the adverse impact of speckle noise on the laser microscope imaging capability as shown in Fig. 3d, e. When the device was not activated (Fig. 3b) the speckle noise corrupted the images making it difficult for the edge detection algorithm to correctly identify both the shapes and the numbers (Fig. 3d). However, when the LC-SR was switched on (Fig. 3c), the edge detection correctly extracted and identified both the shapes and the numbers (Fig. 3e). Line scans along the images in Fig. 3b, c (leading to Fig. 3f, g, respectively) show the improvement in uniformity in the intensity distribution when the LC-SR was turned on, as can be seen by the three fringes that are equally separated with the same thicknesses (Fig. 3g). The intensity of the light was also uniform across the same group of elements, and we can see this from the flat and uniform profile of the plot. These results demonstrate that with the inclusion of the LC-SR the signal to noise ratio of the laser microscope system had noticeably increased.

### LC speckle reducer in a Head-up Display

**Fig. 4:**
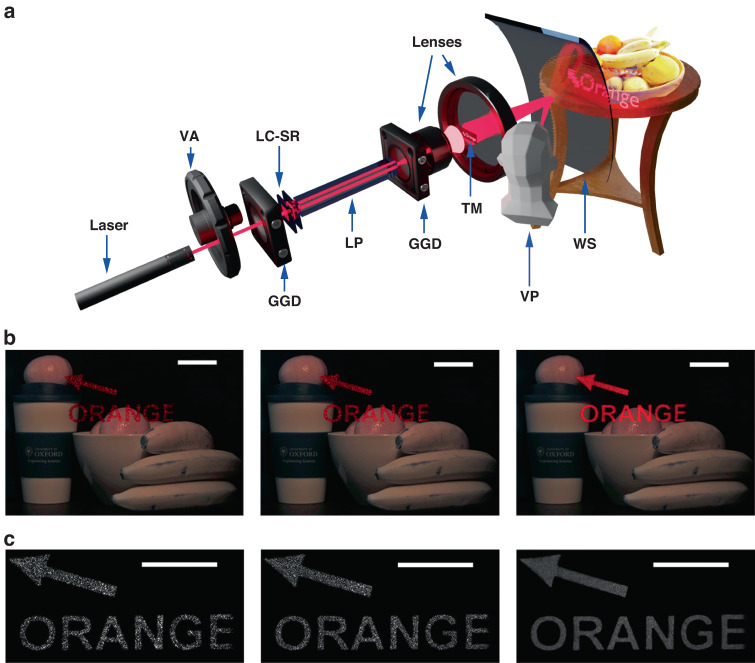
Zwitterionic-doped chiral nematic LC speckle diffuser in a Head-up Display (HUD). **a** Schematic of the HUD demonstrator. The assembly consisted of a variable attenuator (VA), two ground glass diffusers (GGD), a light pipe (LP), the LC speckle reducer (LC-SR), lenses, a target mask (TM) to generate the text, and a windshield (WS). A CCD was positioned at the location of the viewer (viewing point—VP) to capture images of the objects behind the WS as well as the labels that are projected onto the WS. **b** color CCD images of the HUD when there was no LC-SR (left image), with the LC-SR but not operated at the optimum electric field conditions (middle image), and with the LC-SR operated at the electric field conditions corresponding to the lowest speckle contrast value (right image). **c** Monochrome and enlarged images of the HUD when there was no LC-SR (left image), with the LC-SR but not operated at the optimum electric field conditions (middle image), and with the LC-SR operated at the electric field conditions corresponding to the lowest speckle contrast value (right image). The scale bars in (**b**) and (**c**) represent 5 cm. The best performing LC-SR was employed for this demonstration (BL006 + 3.0 wt.% R5011 + 0.5 wt.% Reichardt’s dye, *d* = 40 μm operated at a temperature of 50 °C—corresponding to a speckle contrast of *C* = 0.07)

Another application where lasers are desirable illumination sources, but the presence of speckle noise can corrupt images, is a HUD^49–51^. For this investigation we assembled the system shown in Fig. 4a where a curved piece of plastic was used to mimic an automotive windshield (WS). A bowl of fruit and other objects were placed behind the windshield so that they would be visible to the observer. At the same time, labels and signage were displayed on the windshield (using a target mask (TM) placed before the windshield) that appeared overlayed on the objects at the same focal plane to simulate augmented reality (in this case the observer was provided with details about what the object is, i.e., an orange).

As can be realized from the CCD camera images in Fig. 4b, c, the LC-SR significantly improved the quality of the images. The granular noise was suppressed in accordance with the reduction in the speckle contrast and the intensity distribution across the image became more uniform when the electric field conditions approached those that corresponded to the lowest speckle contrast. This resulted in the content of the display being more intelligible against the background objects for the same average image intensities. For a practical implementation of a HUD, either in the automotive industry or for an augmented reality headset, the accurate transfer of information from the display to the observer is of paramount importance. Even though there was some drop in the transmission in accordance with Fig. 2 when the LC-SR was activated, which was compensated for by the variable attenuator, the marked improvement in image clarity is notable. Speckle present in the images can cause a loss of definition and make the projection uncomfortable to view and, critically, can obscure important information that needs to be relayed to the viewer (e.g., the driver of an automotive vehicle). Therefore, the reduction of speckle plays a crucial role in enabling laser illumination with these devices despite the drop in transmission.

### LC speckle reducer in holography

**Fig. 5:**
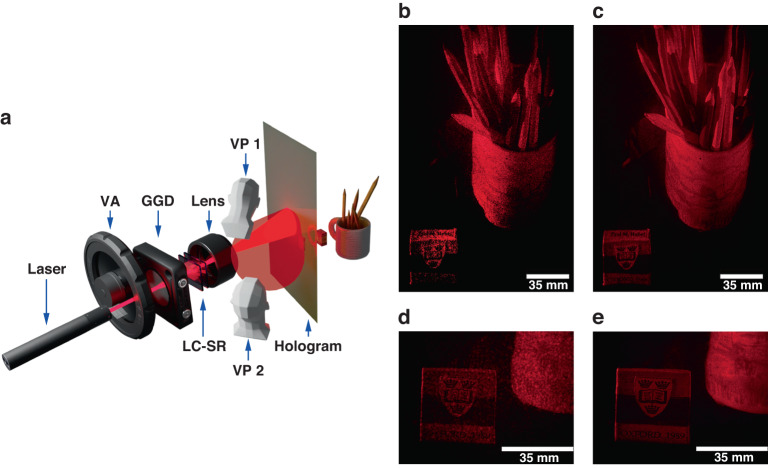
A zwitterionic-doped chiral nematic LC speckle reducer (LC-SR) for holography. **a** Schematic of the experimental assembly used to test the LC-SR with the laser illumination of a thin-film hologram. The system consisted of a He-Ne laser, a variable attenuator (VA), a ground glass diffuser (GGD), the LC-SR and a combination of lenses to expand the beam to illuminate the hologram. the beam is then transported through a positive lens and then projected on a hologram. A CCD camera was positioned at the locations VP1 and VP2 so that the mug and the eraser could be viewed from different angles. **b** Image of the hologram when no LC-SR was included in the system and when the CCD camera was placed at VP1 (positioned high up and at 80 cm from the hologram). **c** Image taken from VP1 when the LC-SR was inserted and operated at the optimum electric field conditions. **d** Image captured on the CCD when it was located at VP2 (positioned low down and close to the hologram) with no LC-SR included in the system. **e** Image captured from VP2 when the LC-SR was inserted and run at the optimum electric field conditions. The best performing LC-SR was employed for this demonstration (BL006 + 3.0 wt.% R5011 + 0.5 wt.% Reichardt’s dye, *d* = 40.0 μm operated at a temperature of 50 °C—corresponding to a speckle contrast of *C* = 0.07)

Speckle can also be an issue in the formation of images in holography^32,52^. Here we demonstrate the performance of the LC-SR when it was used to reduce the presence of speckle in the laser illumination of a multi-level phase hologram, the results of which are presented in Fig. 5. In this demonstration, the output from a He-Ne laser passed through the LC-SR and was then projected on to a hologram that contained 3D information of a cup of pencils and an eraser. We observed the hologram from two different angles: one that was located high up and 80 cm from the hologram (VP1), to see as much of the replay image of the hologram as possible (Fig. 5b, c), The second position was located low down and closer to the hologram (VP2), in order to see the fine detail in the replay image of the hologram (Fig. 5d, e). With the 0.5 wt.% zwitterionic-doped chiral nematic LC-SR subjected to the electric field conditions that lead to the minimum speckle contrast, the appearance of speckle is greatly suppressed. For example, the text and crest on the eraser as well as the pattern on the mug were largely indiscernible without the LC-SR. However, when the LC-SR was inserted and switched on (corresponding to a reduction in the speckle contrast to *C* ≈ 0.07) the logo and text in the image were then accurately reconstructed.

### LC speckle reducer in laser projection displays

**Fig. 6:**
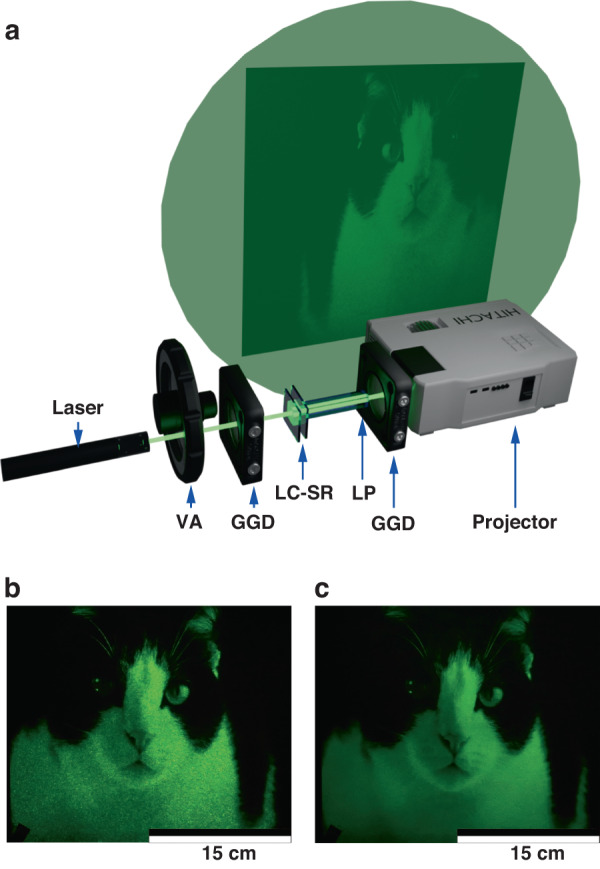
Laser projection with a zwitterionic-doped chiral nematic LC speckle reducer (LC-SR). **a** Illustration of the laser projector demonstrator. The demonstrator consisted of a HITACHI CP-X250 Multimedia LCD Projector that was illuminated by a Quantel laser. Also included were ground glass diffusers (GGD), the LC-SR, and a light pipe (LP). **b** A still from the video of the cat when the LC-SR was not switched on. **c** A still from the video when the LC-SR was operated at the optimum electric field conditions. The video of the cat was downloaded from a copyright-free website (https://pixabay.com). The best performing LC-SR was employed for this demonstration (BL006 + 3.0 wt.% R5011 + 0.5 wt.% Reichardt’s dye, *d* = 40.0 μm operated at a temperature of 50 °C—corresponding to a speckle contrast of *C* = 0.07)

Laser projectors are particularly desirable for cinema and home entertainment systems as they can provide a high brightness with a potentially wide color gamut^1^. For the demonstration of laser projection, we removed the lamp from a HITACHI CP-X250 Multimedia LCD Projector and replaced it with a green laser (Quantel ELBAC-L-559-2-P-M-S-2.0 laser) which emitted at a wavelength of *λ* = 559.6 nm and had a maximum output power of 2 W. A schematic of the laser projector system is presented in Fig. 6a (see Supplementary Information for further details). Examples of the projected image when the laser was operated at its maximum output are shown in Fig. 6b, c when the LC-SR was either not switched on or was run at its optimum electric field conditions. When the LC-SR was not switched on, it can be seen clearly that the image quality of the white fur on the cat was very grainy. Features such as the whiskers were also more difficult to distinguish because of the presence of the speckle. On the other hand, when the LC-SR was switched on, the image quality improved dramatically, and the smooth white fur could be seen clearly without being corrupted by a grainy speckle pattern. A real-time video of the laser projector demonstrator can be found in Supplementary Movie 1. It should be noted that the driving frequency of the LC-SRs did not impact the image refresh rate of 60 Hz as it influences the illuminating light not the formation of the image; this is discussed in more detail in the Supplementary Information.

### LC speckle reducer for laser-based vectorial imaging

The previous sections have demonstrated the performance of the LC-SR in scalar field illumination systems. However, we have also considered how the LC-SR performs when measurements of the vectorial information of light are needed^53–55^. In the following we consider a transmissive geometry (for thin samples under investigation) and then a back-scattering geometry (for a bulk sample). For the transmissive geometry, using Mueller Matrix (MM) imaging, vectorial information of light passing through a thin birefringence crystal was extracted enabling properties such as the retardance and fast axis orientation to be determined^53,56^. The results are also benchmarked with that obtained using an LED illumination source. For the back-scattering geometry, we have validated the vectorial information reconstruction of a bulk porcine liver sample, which is a highly scattering medium, via back scattering illumination. The MMs and their transformation parameters^11,53^ are taken from previous work, through which we successfully retrieved the fine liver polygon structure.

**Fig. 7:**
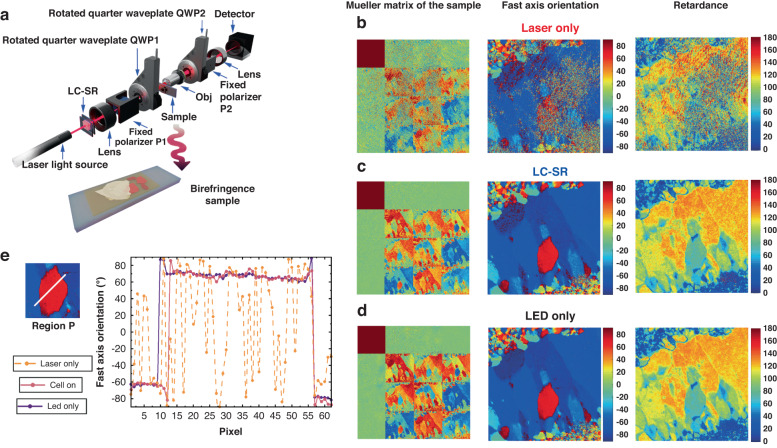
Improving laser-based vectorial imaging of thin-film samples using a zwitterionic-doped chiral nematic LC speckle reducer (LC-SR) (transmissive configuration). **a** Illustration of the transmissive vectorial measurement system and the birefringent sample used in this experiment. **b** MM (left image), fast axis orientation (middle image) and retardance (right image) of the sample for the laser illumination case without the LC-SR. **c** MM image (left image), fast axis orientation (middle image) and retardance (right image) of the sample for the laser illumination case with the LC-SR operated at the optimum electric field conditions. **d** MM image (left image), fast axis orientation (middle image) and retardance (right image) of the sample for the case when an LED (incoherent) is used as the illumination source. **e** An enlarged image of the fast orientation axis of the crystal defined as P extracted from the LED illumination case and a plot of the fast axis orientation as a function of pixel number for all three illumination scenarios over the distance represented by the white solid line in the crystal region defined as P. The best performing LC-SR was employed for this demonstration (BL006 + 3.0 wt.% R5011 + 0.5 wt.% Reichardt’s dye, *d* = 20.0 μm operated at a temperature of 50 °C—corresponding to a speckle contrast of *C* = 0.09)

**Fig. 8:**
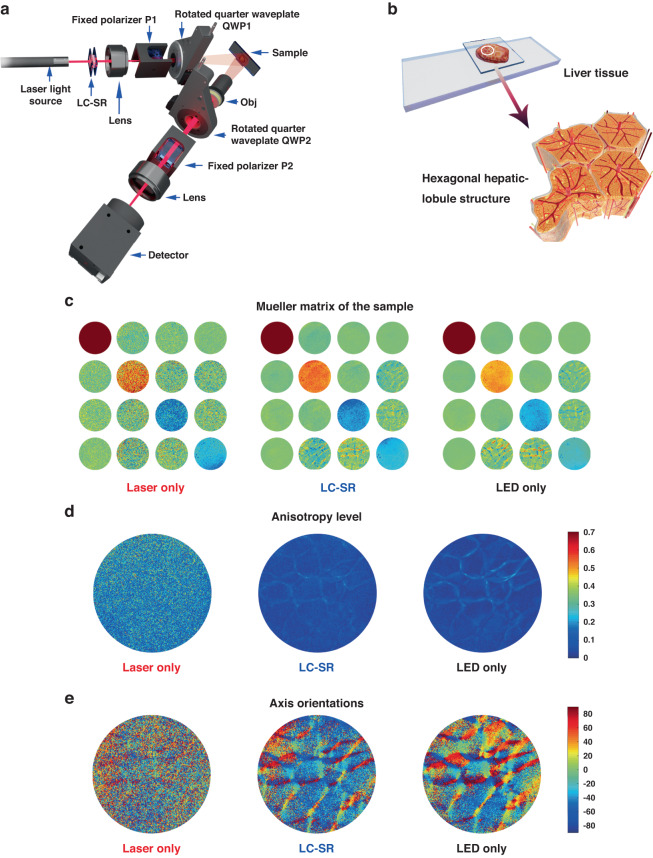
Improving laser-based vectorial imaging of thick porcine liver samples using a zwitterionic-doped chiral nematic LC speckle reducer (LC-SR) (back-scattering configuration). **a** Illustration of the back-scattering vectorial measurement system. **b** Illustration of the porcine liver sample used in this experiment. The hexagonal structure observed in d and e are hepatic lobule structures, which is the basic unit of a liver tissue. **c** Mueller matrices determined for the laser-only illumination case (left image), the laser with LC-SR (middle image) and the LED-only (right image). **d** The level of anisotropy for the laser illumination case (left image), the laser with LC-SR (middle image) and the LED (right image). **e** The orientation of the anisotropy axis across the sample for the laser illumination case (left image), the laser with LC-SR (middle image) and the LED (right image). The best performing LC-SR was employed for this demonstration (BL006 + 3.0 wt.% R5011 + 0.5 wt.% Reichardt’s dye, *d* = 20.0 μm operated at a temperature of 50 °C—corresponding to a speckle contrast of *C* = 0.09)

Figures 7 and 8 showcase the performance of the LC-SR in transmissive and back-scattering vectorial imaging applications, respectively. These figures include illustrations of the vectorial measurement systems, images of the target samples, and the experimental results in the form of the MMs, polarization parameters for three different scenarios: (a) laser illumination without the LC-SR (normal coherence light-based imaging); (b) laser illumination with the LC-SR (speckle reduced coherence light-based imaging); and (c) LED illumination (standard measurement approach). Details of the system configurations, sample information, as well as the MM polar decomposition and transformation processes can be found in the Supplementary Information.

For the transmissive configuration (Fig. 7), we chose one region of the sample to illustrate the performance of the imaging system for the different illumination scenarios. These birefringent samples consist of crystals with different shapes and orientations of the fast-axes. Measurements of the vectorial information of light upon passing through the birefringent sample enable the retardance values and orientation of the fast axis to be determined. These results are presented as colormaps where the legend on the righthand side of the images shows the relationship between the color and the value of either the fast axis orientation (middle image) or the retardance (right image). Considering the data for the MM (first image in Fig. 7d) for LED illumination as an example, it can be seen that the bottom right 3 × 3 sub-matrix includes information about the spatial variation of the birefringent properties of the sample, which demonstrates that the target sample exhibits a strong optical anisotropy^11^. After implementing a polar decomposition method^56^ (see Supplementary Information), a quantitative value for the fast axis orientation (middle image in Fig. 7d) and the spatially varying retardance (right image in Fig. 7d) could be extracted.

In contrast, when a CW He-Ne laser was used as the illumination source (Fig. 7b), we can see clearly that the speckle noise severely affects the intensity distribution leading to significant measurement errors. The MM polarimetry requires multiple intensity measurements to enable the vectorial information to be extracted. If there is significant noise in the intensity, then this degrades the precision of the MM polarimetry^53^. We can see from Fig. 7b that the MM images (left image) are very blurred, with lower values in the bottom right 3 × 3 sub-matrix, as well as unwanted noise appearing in the first row and column of the MM. In addition, the quality and accuracy of the deconstructed fast axis orientation (middle image) and retardance (right image) are in turn corrupted by the noise present in the intensity, which results from the presence of speckle.

From these images we have identified a crystal region (defined by P) that has a very distinguishable fast axis orientation relative to the surrounding background and have used this to enable a quantitative comparison of the performance for the three different illumination cases. The crystal defined by P clearly has a very different orientation (which appears red in our colormap) compared with the surrounding region (which appears blue in our colormap). When using just the laser as an illumination source the region defined by P cannot be readily identified (Fig. 7b—middle image). In contrast, however, with the assistance of our LC-SR device, the full MM images (Fig. 7c—left image) as well as the fast axis orientation (Fig. 7c—middle image) and retardance (Fig. 9c—right image) are accurately recovered and are in good agreement with the standard incoherent (LED) illumination case (Fig. 7d).

Figure 7e plots a value for the fast axis orientation across the region defined by the white line in the image where using laser illumination the fast-axis value (dashed orange curve) oscillates dramatically across the sample showing no clear signs of a crystal structure. This is in contradiction to the actual sample properties as reflected in the results for the standard LED illumination (blue solid line) and when our LC-SR is combined with the laser source (solid red line). Encouragingly, the results obtained for our LC-SR agree extremely well with those obtained for the LED illumination scenario. Considering the unique properties of a laser light source, such as (1) high intensity, (2) narrow bandwidth and (3) compatibility with a fluorescence microscope, a laser light source would be beneficial over an LED, especially with speckle suppression.

For the back-scattering configuration (Fig. 8a), a porcine liver tissue sample was selected for this demonstration (Fig. 8b). The liver is a very important organ, which performs hundreds of different functions including lymph production, protein metabolism and hormone production^57^. These complicated physiology functions are closely related to the intrinsic microstructure of the liver tissue and the abundance of organelles within cells^58^. MM imaging is an excellent label-free technique for distinguishing the microstructural variations in the surface layer of the liver in order to help diagnose hepatic diseases^59,60^. Previous studies have shown that the porcine liver is almost isotropic^59,60^. However, there exists polygonal structures distributed around the isotropic liver tissues, which are highly birefringent connective tissues and are considered to be the boundaries of hepatic lobules^61^. Monitoring such anisotropic structures is beneficial in terms of the detection and diagnosis of the liver condition^59,60^.

As before, results are presented (Fig. 8) for the three illumination scenarios: laser on its own, laser with LC-SR, and incoherent illumination using an LED. The latter represents the traditional approach and helps to benchmark the performance of the speckle reducing technology presented herein. Using MM polarimetry (Fig. 8c), the level of optical anisotropy (Fig. 8d) as well as the anisotropy axis orientation (Fig. 8e) can be determined—(see Supplementary Information for further details). It is worth noting that here the back-scattering bulk tissue is an intense light scattering media, and previous laser-based full MM vectorial imaging has not been able to retrieve the fine polygonal structures shown here (for the laser illumination similar cases have been considered in ref. ^62^). Using our LC-SR device, the influence of laser speckle can be significantly reduced enabling accurate reconstruction of the intricate polygonal structures of the liver tissue (see the middle images in Fig. 8c, d, e). To the best of our knowledge, this is the first time the image quality as well as the vector information obtained from back-scattering MM polarimetry has been recovered with laser illumination and low speckle *C* = 0.09.

## Discussion

The above observations show that the zwitterion doped LC-SR device made a significant improvement for laser-based scalar imaging and vectorial imaging—in both image resolution and the vectorial information correctness—via its sophisticated speckle-reduction. We demonstrated that upon the addition of the 0.5 wt.% Reichardt’s dye to a chiral nematic LC (BL006 + 3.0 wt.% R5011), the speckle noise can be reduced from *C* = 0.7 ± 0.02 to *C* = 0.07 ± 0.02 for a temperature of 50 °C with a 40 μm-thick cell. These 40 μm-thick devices also exhibited a very low speckle contrast of *C* = 0.075 at a temperature of 25 °C enabling applications operating at room temperature.

The investigation of the LC-SR is not limited to device research. We proved that the ability of our device is not dependent on the specific applications but can be adopted to various configurations ranging from light-field microscopy, HUD, holography to laser projector. The results and performance of the LC-SR in these applications are exciting and promising. Compared with previous work on speckle reduction in an optical microscopic system, Redding et al. proposed a colloidal solution as a speckle reducer that could reduce the speckle contrast from *C* = 0.05 to *C* = 0.03 (resulting in an approximately 40% suppression)^63^. For holography applications, techniques are typically divided into two categories: (1) optical approaches and (2) numerical approaches. In relation to the first methodology, recent work has demonstrated a reduction from *C* = 0.44 to *C* = 0.08 of a reconstructed USAF image from a SLM digital hologram in the Fraunhofer zone with partially coherent illumination^64^.

Apart from demonstrations that involve the scalar field nature of light, this is the first time that a speckle reducer has been used in a vectorial imaging system with such good performance as well as leading to promising results and comparisons. Previously, a study has been conducted using an electroactive polymer-actuated speckle reducer in a MM polarimetry experiment to observe cartilage explants in a backscattering configuration. However, the report only considered the speckle contrast results for a Spectralon surface with and without the speckle reducer. It did not include a comparison of the MM measurements of the specimen for a laser light source. The reported speckle contrasts for the Spectralon surface were found to be 25.1 ± 0.4% and 60 ± 0.2%^62^, with and without the speckle reducer, respectively. Another study also employed a commercial speckle reducer (LSR-3010 Series, Optotune, Dietikon, Switzerland) in a MM measurement system to investigate inflammatory skin diseases in a backscattering configuration. However, this work did not quantify or compare the performance of the device after it was inserted into the MM acquisition system^65^. The exciting results shown in Figs. 7 and 8 emphasizes the possibility of improving the resolution of fluorescence microscopy which is an essential imaging technique in fluorescence polarization microscopy^66,67^ that suffers from the speckle noise due to the coherent nature of the laser light. With this LC-SR, the use of laser light provides a narrow bandwidth as well as high intensity illumination enabling the image resolution and vector information correctness to be significantly enhanced in the vectorial domain. The LC-SR device shows good performance for different microscopy configurations encompassing scenarios ranging from the analysis of birefringent materials to the characterization of thick tissue biopsy.

The LC-SR demonstrated herein is compact, portable and has a low form factor. The small dimensions of the device make the integration into existing commercial and custom-built systems that currently suffer from speckle noise relatively straightforward. The applications we presented here cover a broad range of imaging/display systems and extends the potential application further to more sophisticated vectorial imaging. The notable change and suppression of the granular noise resulting from the LC-SR demonstrates the capability of such technology for the improvement of laser microscopy, HUD, holography, laser projector and polarization microscopy.

## Materials and methods

### Mixture preparation and device fabrication

The base chiral nematic LC was composed of the nematic LC mixture, BL006 (Merck), and the high twisting power chiral dopant (R5011). The base mixture followed the same procedure reported in the previous work^39^. Reichardt’s dye was chosen from various zwitterion dopants and the concentration of the dopant was carefully investigated in order to determine the best performance in terms of speckle reduction. The mixture was filled via capillary action into commercially available INSTEC LC2 cells that have a nominal cell gap of *d* = 20.0 μm, or home-made cells with a gap of *d* = 40.0 μm. The home-made thicker glass cells (*d* = 40.0 μm) had an anti-parallel alignment layer coated onto the top and bottom indium tin oxide (ITO)-coated glass substrates after 10 min of washing in an ultrasonic bath. The two ITO glass substrates were separated by 40 μm thick spacer films.

The concentration of the chiral dopant R5011 was carefully chosen based upon previous studies and the resulting pitch (*p*) was estimated from the reflection band using a UV-vis spectrometer (Agilent Cary 8454). There are no nano or micro-particulates in these LC devices as they contain a chiral nematic LC and zwitterion dopant molecules only. Therefore, one of the key length scales that influences the degree of scattering is the pitch of the chiral nematic LC helix. As a result, this parameter was controlled and characterized for all of the devices produced. Supplementary Information Fig. S1 shows how the speckle contrast changes with the pitch as well as the zwitterion dopant concentration. The best speckle suppression can be realized for a pitch of *p* = 310 ± 10 nm where the concentration of the chiral dopant R5011 is about 3.0 wt.%. The trend is consistent for different concentrations of the zwitterion dopant Reichardt’s dye. Therefore, the base mixture that we chose for this study consisted of the nematic LC BL006 and 3.0 wt.% R5011. The concentration of Reichardt’s dye is discussed in more detail in the main text and the Supplementary Information.

### Characterizing laser speckle

For characterizing the speckle contrast reduction with the LC-SRs, a Helium-Neon (He-Ne) laser first passed through a homogenization system consisting of a light pipe contained between two ground glass diffusers. The LC-SRs were placed between the first diffuser and the light pipe when characterized and were held in a mount with a hot-stage so that they can be held at a constant temperature. The end of the homogenization system was then imaged onto a paper screen to form a square of uniform intensity thereby eliminating the underlying gaussian intensity distribution of the laser beam. A CCD camera was used to capture a section of this image in order to calculate a corresponding speckle contrast value from the intensity distribution. Angular dependence with regards to speckle reduction is more closely tied to the gain of the screen used to display the projected image rather than the speckle reduction device. In this characterization experiment, the screen can be approximated as Lambertian. As such, notable changes in the magnitude of the speckle reduction are not observed when the angle of viewing and/or illumination is altered. The speckle characterization system was designed to minimize these angles in accordance with the Laser Illuminated Projector Association (LIPA)^68^ guidance on measuring speckle in laboratory conditions.

The CCD camera has a bit depth up to 14, which is above the threshold for accurately measuring speckle. Additionally, the camera was cooled to minimize the impact of noise on the measurement, which was operated with unity gamma correction to provide a linear relationship between the pixel value and the incident optical intensity. The focal length of the lens, the aperture for the camera and the integrating time were selected to mimic conditions present in the human eye to ensure speckle values calculated closely match human perception. Additionally, the impact of ambient light was reduced by enclosing the setup and it was ensured that the camera was not saturated during any measurement by installing a suitable neutral density filter in front of the laser. However, the value of the filter was carefully selected to maximize the use of the full dynamic range of the camera. Details of the system configurations in which the LC-SR are showcased are provided in Part VIII of the Supplementary Information.

### Supplementary information

Supplementary Information

Supplementary Video 1
