# Supplementary material for: Zwitterion-doped liquid crystal speckle reducers for immersive displays and vectorial imaging
Source: Light Sci Appl. 2023 Sep 22;12:242. doi: 10.1038/s41377-023-01265-5 (PMC10514055; doi:10.1038/s41377-023-01265-5)
Supplement: Supplementary file 1 — Supplementary Information [file 41377_2023_1265_MOESM1_ESM.docx]

Supplementary Information

Zwitterion-doped Liquid Crystal Speckle Reducers for Immersive Displays and Vectorial Imaging

Yihan Jin, Nathan P. Spiller, Chao He*, Grahame Faulkner, Martin J. Booth, Steve J. Elston*, and Stephen M. Morris*

*Department of Engineering Science, University of Oxford, Parks Road, Oxford, OX1 3PJ, United Kingdom*

Correspondence:

Chao He ([chao.he@eng.ox.ac.uk](mailto:chao.he@eng.ox.ac.uk))

Steve J. Elston ([steve.elston@eng.ox.ac.uk](mailto:steve.elston@eng.ox.ac.uk))

Stephen M. Morris ([stephen.morris@eng.ox.ac.uk](mailto:stephen.morris@eng.ox.ac.uk))

1. **Chiral nematic pitch and zwitterion dopant concentration**


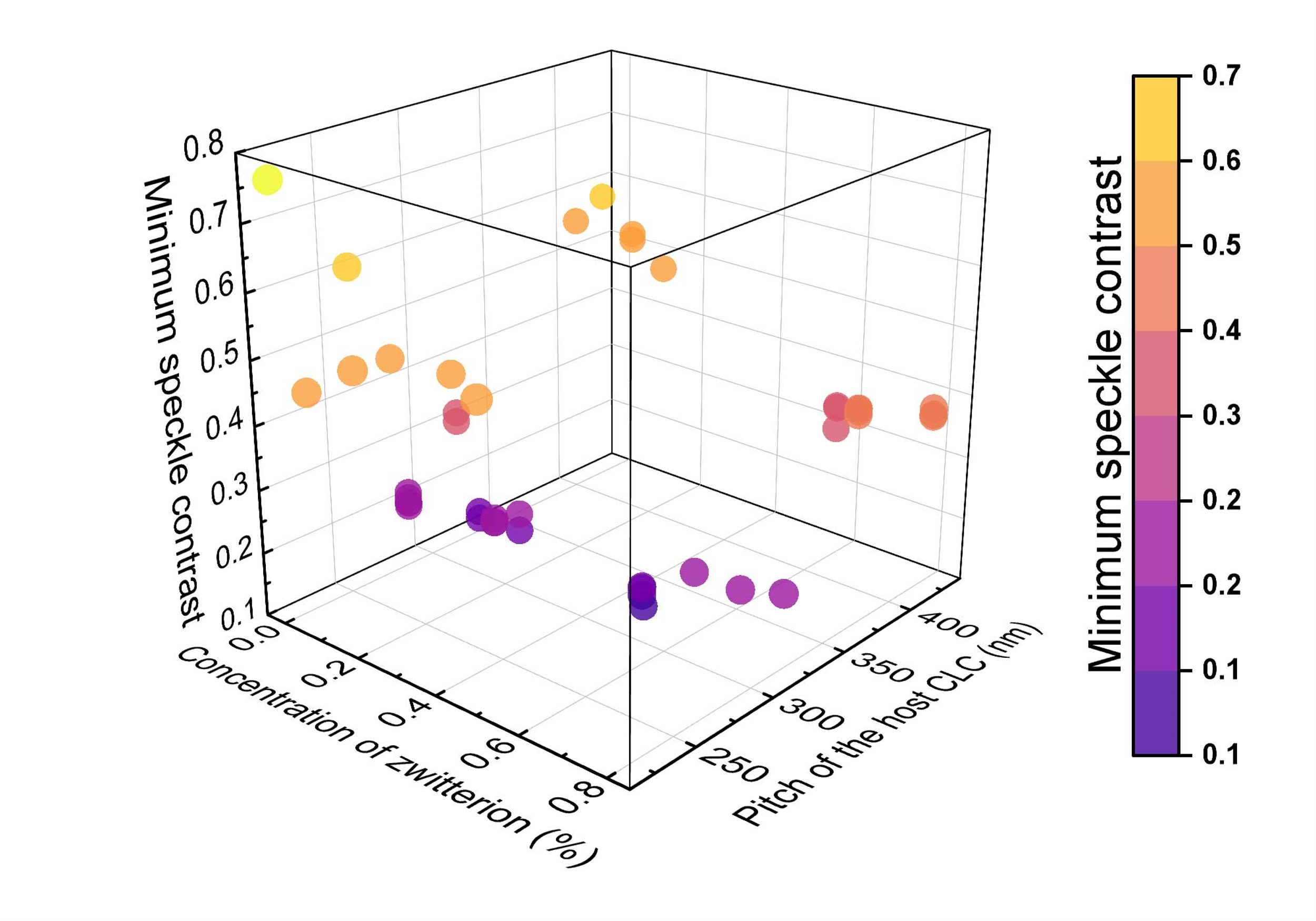


**Fig. S1** The minimum speckle contrast as a function of the pitch of the chiral nematic liquid crystal host and the concentration of the zwitterion dopant.

The data points in **Fig. S1** represent the minimum speckle contrast observed for different pitch values of the chiral nematic liquid crystal (LC) host and the concentration of zwitterionic dopant. The color of the data points indicates the magnitude of the speckle contrast when the cell was operated at optimum voltage driving conditions. The mixture for each test was composed of a nematic LC (BL006, Merck) and chiral dopant (R5011, Merck) ranging in concentration from 2.5 wt.% to 5 wt.% so that a range of pitch values could be tested. Each mixture was filled into glass cells with a gap of 20 μm and operated at room temperature. Fig. S1 shows that the lowest speckle contrast occurs around the region where the concentration of zwitterion in the chiral nematic host is 0.5 wt.% and the pitch is around 310 ± 10 nm. Based on these results, the chiral nematic LC mixture chosen for testing in the different imaging and display applications was BL006 + 3.0 wt.% R5011 + 0.5 wt.% Reichardt’s dye.

1. **Role of the Zwitterionic Dopant**

The dye used in this study is a type of zwitterionic dopant that has an equal number of positively and negatively charged functional groups, which enables the dye to be either an acceptor or a donor. After doping into the LC mixture, a dye molecule can either accept or donate one electron at the cathode or anode, respectively, and then drift to the opposite electrode of the device under an applied electric field. A positive RD molecule (RD+) will accept an electron at one electrode whereas a negative RD molecule (RD-) will discharge an electron at the other electrode, thereby transferring to a neutral dopant. These dopants then diffuse to the opposite electrode where they then convert electrochemically into either an anion or cation.

To maintain a reversible process, two factors are important: 1) the frequency of the electric field cannot be too large otherwise the freshly generated anions and cations would not be able to move to the opposite electrodes; 2) the electron concentration that is governed by charge injection at the electrode, which relates to the amplitude and the frequency of the applied electric field. This indicates that a large electric field would be desirable. However, as the devices need to operate in a dynamic scattering mode due to EHDI, very large electric field amplitudes are not desirable as this would result in an unwinding of the helical structure which would prohibit the formation of dynamic scattering. It is thus expected that higher dye concentrations, which would benefit from larger charge injection and thus higher electric field amplitudes, do not necessarily aid the generation of the scattering mode as the larger electric fields would lead to a transition to the homeotropic nematic configuration, which does not lead to any speckle reduction. Therefore, in this case, additional zwitterionic dye molecules (corresponding to an increase in the concentration of the zwitterionic dye) would not be able to participate in the electrochemical reactions and hence generate more turbulence.

The speckle contrast colormap in Fig. 1b shows that there is a transition in the speckle performance at a particular amplitude of the electric field that appears relatively independent of the frequency. This can be explained as follows. For the neat chiral nematic LC mixture (i.e., without any zwitterionic dopants, Fig. 1b) there are only a small number of ions present because of the natural impurity of the mixture. In such devices, the threshold electric field for unwinding the chiral nematic LC into a homeotropic nematic is the primary reason for the abrupt change with increasing electric field amplitude.

When the helix is completely unwound, the LC director aligns with the electric field direction, forming a homeotropic nematic LC arrangement. In this case, the mixture is not dynamically changing, and the scattering is minimized hence there is effectively little or no speckle reduction (as indicated by the speckle contrast values). The ionic motion in the device, on the other hand, largely governs the frequency dependence of the performance. For the neat chiral nematic LC mixture in Fig. 1b, there are minimal ions present so there is little in the way of frequency dependence of electric field amplitudes above *E* = 16 V μm^-1^. The transition to a homeotropic nematic alignment at these electric field amplitudes has been verified by polarizing optical microscopy (POM) images which show the LC texture becoming completely dark between crossed polarisers with nearly zero light scattering even over a large range of frequency.

The colormap for the speckle contrast of the zwitterionic-doped LC-SR in Fig. 1d demonstrates the effect the zwitterionic dopant has on the reduction in the speckle contrast for different electric field amplitudes and frequencies. A notable feature is that the transition from the regions where the speckle contrast is reduced (corresponding to an intense dynamic scattering mode) to the regions where the speckle contrast shows no obvious reduction (when the helix has been unwound) appears to be somewhat different from that shown in Fig. 1b. In contrast to that observed for the LC mixture without zwitterionic dopants, the mixture with the dopants shows that a transition to the regions where there is no speckle reduction (indicated by the dark red regions) appears to depend upon both the amplitude and the frequency of the applied electric field.

The two factors of importance are: 1) the scattering caused by the chiral nematic LC and the turbulence of the microdomains and 2) the motion of the zwitterionic dopants. The first factor is mainly affected by the amplitude of the electric field. The degree of the scattering as well as the motion of the chiral nematic microdomains decrease when the electric field increases. The second factor, the motion of the ions, depends upon both the amplitude and the frequency of the applied electric field. Electric field conditions that do not match the optimum conditions will cause a decrease in the turbulence resulting in a decrease in the speckle reduction, which is why the transition to the regions where there is no speckle reduction appears to be dependent upon both the electric field amplitude and the applied frequency in Fig. 1d.

1. **Saturation Electric Field of Liquid Crystal Speckle Reducers**

**
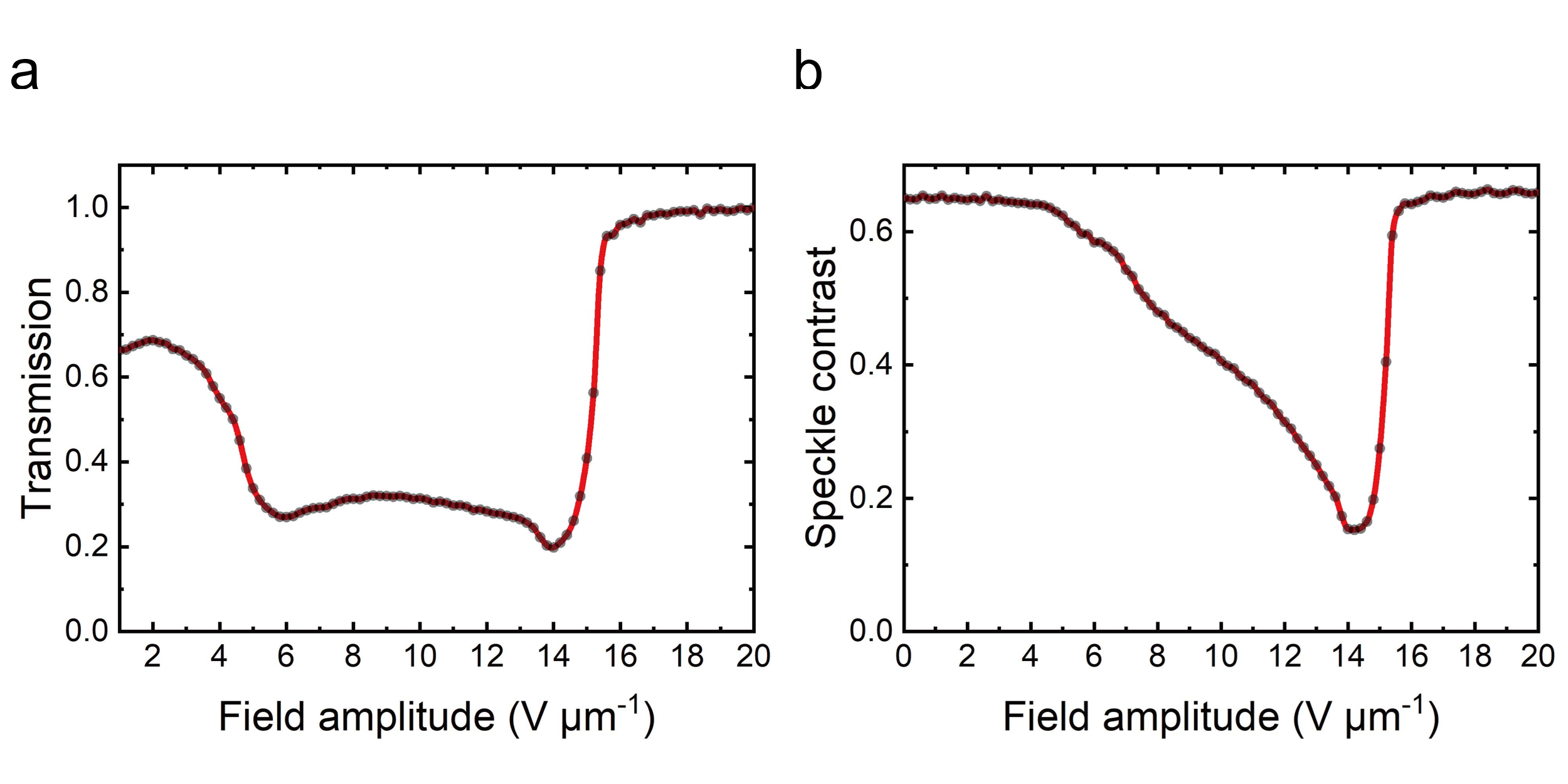
**

**Fig. S2** Dependence of **a** transmission and **b** speckle contrast on the electric field amplitude for the LC speckle reducer presented in Fig. 1c.

To determine the electric field for which both the speckle contrast and the transmission were found to saturate (for the LC-SR presented in Fig. 1c), the optimum operating frequency (as identified in Fig. 1c) was selected. The electric field was then systematically increased from *E* = 0 V µm^-1^ to *E* = 20 V µm^-1^, in steps of 0.2 V µm^-1^, while maintaining an applied frequency of *f* = 26 Hz. As the electric field increased incrementally, the transmission was found to gradually decrease until reaching a minimum at *E* = 14 V µm^-1^ (**Fig. S2**a). Subsequently, as the electric field amplitude was increased further the transmission increased rapidly before reaching saturation at a critical electric field of *E_c_* = 16 V µm^-1^. This saturation in the transmission is also reflected in the dependence of the speckle contrast. As Fig. S2b clearly illustrates, the speckle contrast rapidly increased with electric field amplitude, reaching a saturation in the speckle contrast (one that becomes independent of electric field) above values of *E_c_* = 16 V µm^-1^, in accordance with the results for the transmission presented in Fig. S2a.

1. **Observation of the Dynamic Scattering Mode**

**Fig. S3**a-c illustrate the director configuration of the chiral nematic LC in the glass cell before being subjected to an electric field (**Fig. S3**a) as well as for the cases when the electric field is applied (Fig. S3b) and then subsequently removed (Fig. S3c). Before the LC devices were first subjected to an electric field, the director formed a Grandjean texture (helical axis aligned parallel to the normal of the substrates). When the optimum electric field conditions for speckle reduction were applied to the LC device, a dynamic scattering state was formed due to EHDI. In this state, the director was randomly oriented in space and time because of the dynamic flow generated by the movement of the ions. Subsequently, when the electric field was removed the flow inside the device subsided (which usually takes 1-2 seconds), the disordered director became time invariant, forming a static focal conic scattering state. If left undisturbed for a longer period (days/weeks) this static focal conic texture would slowly revert to a more Grandjean configuration.


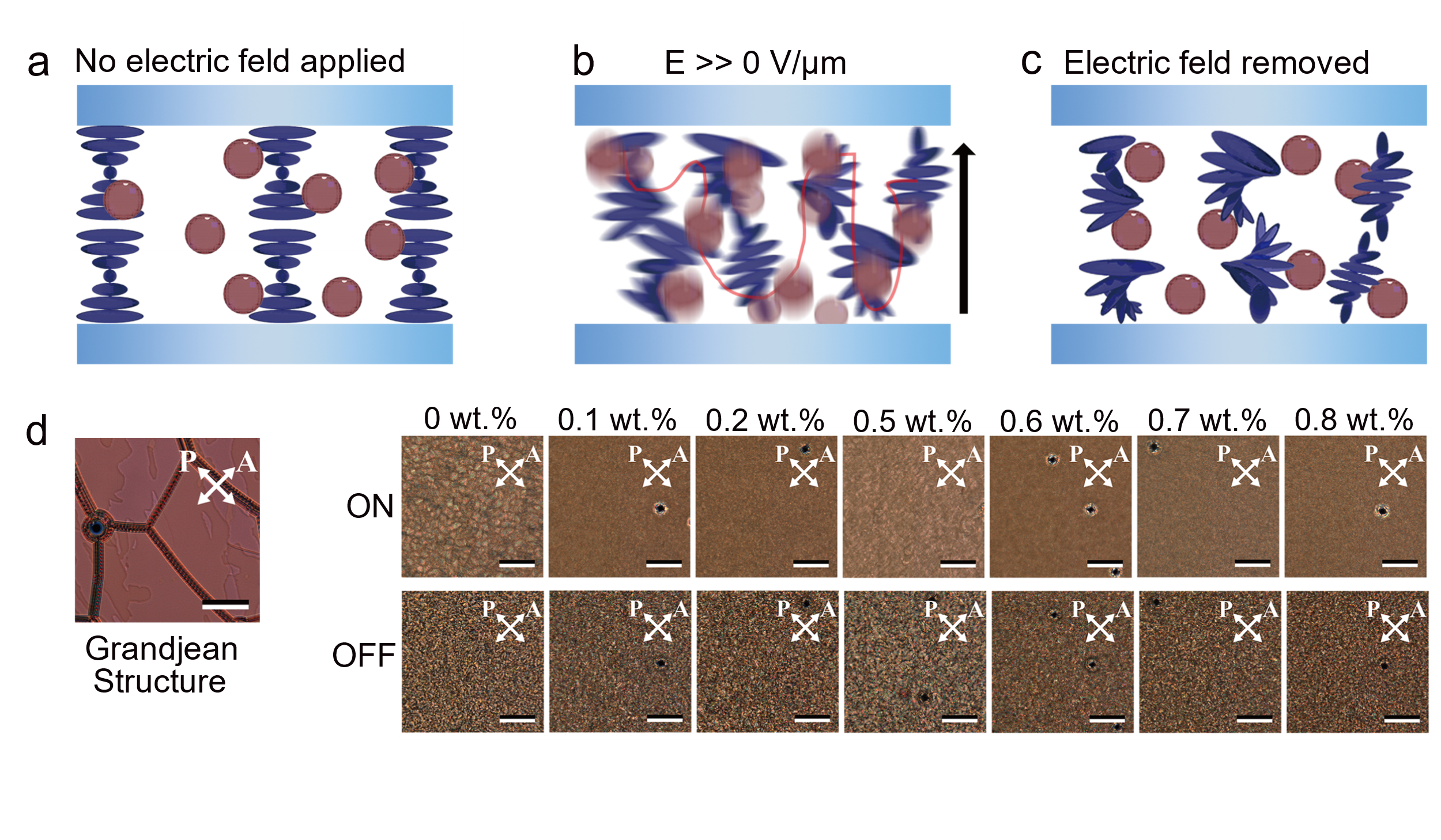


**Fig. S3** **LC director configuration and polarizing optical microscope (POM) images of the LC-SRs under different electric field conditions**. Illustrations of the LC director configuration of the chiral nematic doped with the zwitterionic dopant: **a** before the device is first exposed to an electric field; **b** under optimum electric field conditions resulting in a dynamic scattering mode; **c** when the device is switched off after applying an electric field, resulting in a static scattering state. The blue prolate shapes represent the LC director and the red spheres are the zwitterionic dopant. **d** POM image of the Grandjean texture before an electric field applied and POM images of the LC-SRs with and without the optimum electric field conditions applied for different concentrations of the zwitterionic dopant (shown above the images). The devices consisted of LC layers that were *d* = 20 μm and the images were taken at 25 °C. Scale bars in the POM images are 50 μm.

The corresponding POM images of the scattering states for the LC mixtures with different concentrations of the zwitterionic dopant with the same LC layer thickness of 20 μm, are shown in Fig. S3d. Before the electric field was applied, the chiral nematic LC adopted the familiar Grandjean texture as a result of the planar alignment layers. When the electric field was applied the degree of turbulence and the size of the dynamic scattering domains varied depending upon the concentration of the zwitterionic dopant (top row of POM images). For the device without a zwitterionic dopant, the optical structure observed when the electric field was applied appeared to be a combination of stationary focal conic domains and a dynamic scattering mode. However, upon the addition of zwitterionic dopant, the dynamic scattering domains dominate and appear to be optimized at a concentration of 0.5 wt.%. When the electric field had been removed (bottom row of images), the chiral nematic LC adopted a stationary focal conic alignment as can be seen by the optical textures in the images. Variants of this optical texture were observed in cells with different concentrations of zwitterionic dopant, and it is believed that this static configuration was largely governed by the pitch of the chiral nematic LC host, the cell gap, and the alignment layers.

1. **Scattering Haze**

For measurements of the haze, the laser light first passed through an attenuator to make sure the detector was not saturated before it then propagated through the LC-SR device. The transmitted laser light was then collected and measured by a photodiode placed 5.5 cm behind the LC-SR. The intensity when there was no light present on the detector was measured and treated as an offset, which was then deducted from all subsequent measurements. The reference haze value was taken when each LC-SR device was subjected to large electric field amplitudes to unwind the chiral nematic helix.

Values for the scattering haze (*H*) associated with the three different devices (see Fig. 1b-d) exhibit comparable trends and transitions to those observed in the speckle contrast colormaps. Results for the haze are shown in **Fig. S4**, which are also presented in a colormap format with values ranging from *H* = 0 to 1, whereby *H* = 0 indicates no haze (and so the device is transparent) to *H* = 1 when the device is highly scattering. For the chiral nematic LC device without any zwitterionic dopant (Fig. S4a), the haze value was found to decrease from *H* = 0.97 (the red dashed ellipses in the haze colormap) to *H* ≈ 0 (at *E* = 17 V µm^-1^, *f* = 25 Hz). Similar reductions in the haze are also demonstrated in Fig. S4b, c. Comparing Fig. S4a with Fig. 1b, when the speckle contrast increased from the lowest value of *C* = 0.15 (the red dashed ellipse) to *C* = 0.33 (at *E* = 15 V µm^-1^, *f* = 26 Hz), the corresponding haze value decreased by 8% (as shown in Fig. S4a).

Considering both the results for the speckle contrast and the haze, the minimum speckle contrast was found to correspond to a haze value of *H* ≈ 1. Conversely, when the macroscopic helix of the chiral nematic LC was unwound, resulting in no speckle reduction, the haze value was found to drop to *H* ≈ 0. These results are consistent with previous reports^1^ where it was stated by Zhang Y *et al.*, that dynamic scattering modes driven by EHDI can result in large haze values. Our observations further indicate a direct correlation between the degree scattering in the LC-SR devices and the resulting decrease in speckle contrast, coupled with an increase in the observed haze.


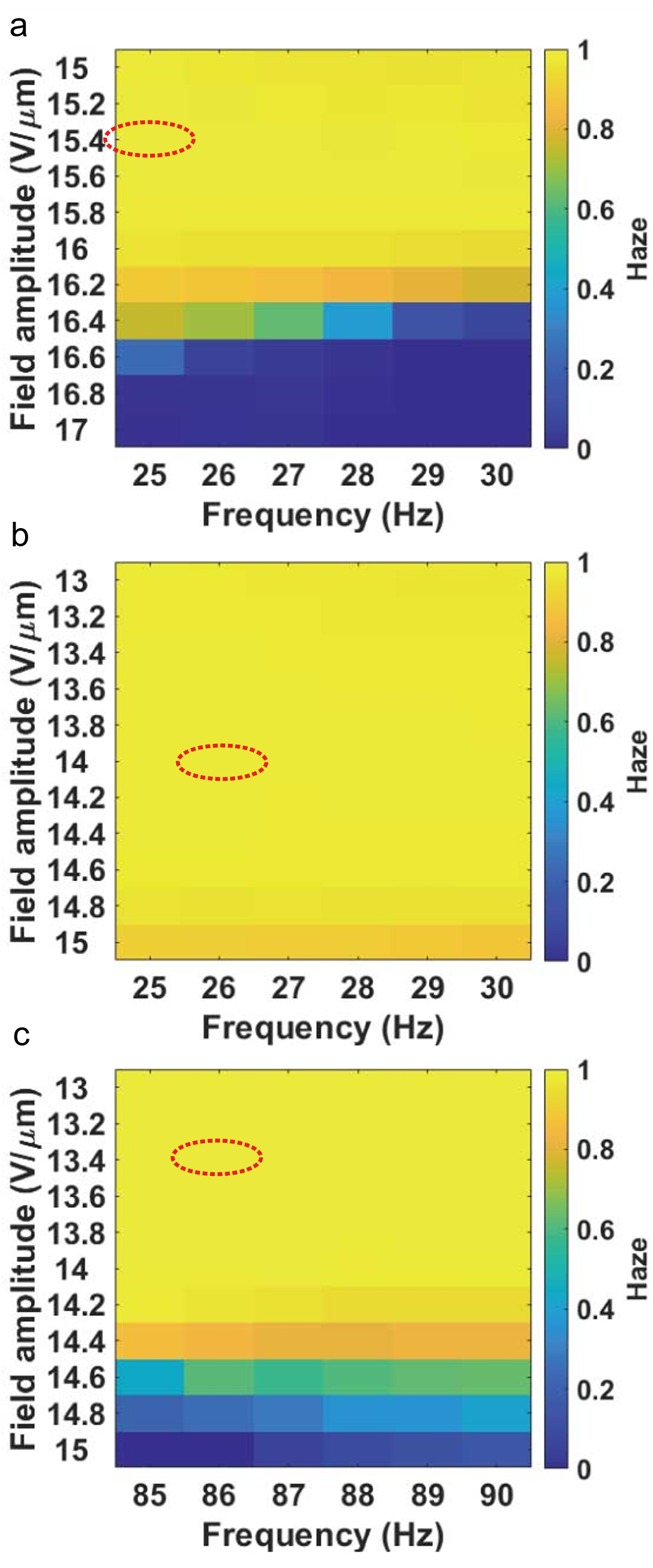


**Fig. S4** **The scattering haze recorded as a function of the electric field amplitude and frequency for three different LC-SR mixtures and devices:** **a** LC-SR consisting of a *d* = 20 μm LC layer at a temperature of 25 °C for the base chiral nematic LC mixture. **b** LC-SR consisting of *d* = 20 μm LC layer at a temperature of 25 °C for the chiral nematic LC mixture with 0.5 wt.% zwitterionic dopant. **c** LC-SR consisting of a *d* = 40 μm LC layer at a temperature of 50 ℃ for the chiral nematic LC mixture with 0.5 wt.% zwitterionic dopant. The red dashed ellipse in each plot correspond to the smallest speckle contrast value recorded in Fig. 2.

1. **Response Time of Liquid Crystal Speckle Reducers**


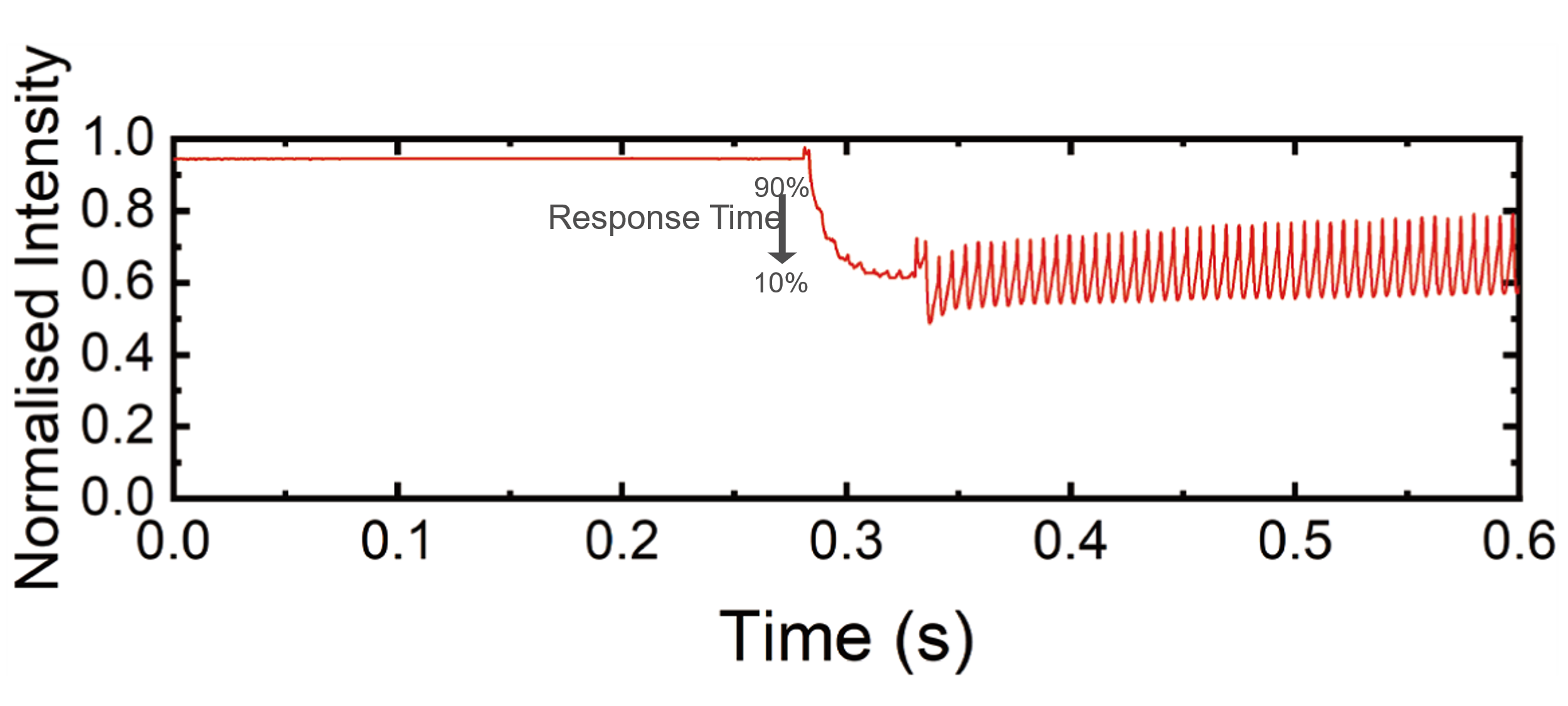


**Fig. S5** Variation in the intensity of light scattered from an LC-SR when the electric field was applied as a function of time.

The plot in **Fig. S5** shows the transient temporal fluctuations in the intensity of light scattered from the 40 µm-thick LC-SR (from Fig. 1d in the manuscript) when the optimum electric field was suddenly applied (just after 0.75 s). These measurements enable an estimate of the response time for the optimum speckle reduction to be achieved from when the optimum electric field was applied to the device. For this application the response time was defined as the standard rise/fall time definition of 90-10 %. The measured value of the response time of our device was found to be approximately 24 ms, and so it should not be the cause of any significant delays in practical systems such as laser projectors or HUDs.

The driving frequency of the LC-SRs did not impact the image refresh rate of 60 Hz as it influences the illuminating light not the formation of the image. For the optimum electric field conditions there are small oscillations/changes in intensity; however, these are not significant enough to induce a noticeable effect for a human observer. The LC-SRs were driven at relatively low frequencies in accordance with the data presented in Fig. 1, showing a combined range of 25 - 90 Hz. However, these are zero-mean squares waveforms which contain higher order harmonics due to their sharp edges. Through Fourier analysis of the intensity fluctuations of the LC-SR (Fig. 1d) in its stable speckle reducing state, the frequency of the fluctuations in the LC optical texture can be estimated. When in the dynamic scattering regime the first prominent frequency component is typically seen at twice the electric field frequency (for the optimum performing 40 µm cell driven at 86 Hz the peak was observed between 172 -175 Hz). This shows the scattering has a dependence on the transitions between positive and negative electric field values for the square wave.

The transition/change in direction of electric field will influence the zwitterion motion, which is a strong contributor to the formation of the dynamic scattering state through EHDI. Further peaks in the spectrum occur regularly and evenly spaced up into the higher order frequencies. Their amplitudes decay steadily up to 4 kHz and then decay much more rapidly rendering them almost negligible by 10 kHz. This provides an estimated upper frequency for the fluctuations in the LC-SRs dynamic scattering texture of around 4 kHz. When the LC-SR was driven away from its optimum electric field conditions the higher frequency components reduced in amplitude. By observing the even spacing of the peaks in the frequency response of the fluctuations in the LC-SR it is reasonable to suggest these are responses in the LC texture corresponding to the higher harmonic frequencies contained within the applied square electric field waveform. As such, this suggests that by providing the required electric field conditions to form a dynamic scattering mode within the device, high frequency fluctuations can be generated in the response corresponding to the higher harmonics in the drive electronics. These high frequency fluctuations are what enable the LC-SRs to reduce speckle considerably even in the short integration time of the human eye.

The speckle statistics can also provide approximations for the frequency in the fluctuations of the LC-SR by approximately equating this to the rate of generation of the decorrelated speckle patterns. The top performing devices were able to reduce the speckle contrast by a factor of 11. From Goodman’s description of generating decorrelated speckle patterns this corresponds to over 120 patterns being generated within the 50 ms integration time of the detector^2^. This implies decorrelated speckle patterns being generated at a rate of over 2 kHz. The observations from the frequency spectrum of the intensity fluctuations match well with this theoretical speckle pattern generation rate. The dynamic scattering mode in the LC-SR will be generating a set of speckle patterns which will contain a subset of completely decorrelated patterns. These are what contribute to the speckle reduction. Therefore, it is expected that the fluctuations present in the LC optical texture should be on the same order of magnitude as the predicted frequency from the speckle statistics. It is also expected to be slightly higher that the decorrelated pattern rate as not all the LC optical textures generated will produce a completely decorrelated speckle pattern.

1. **Edge detection in optical microscope demonstrator**


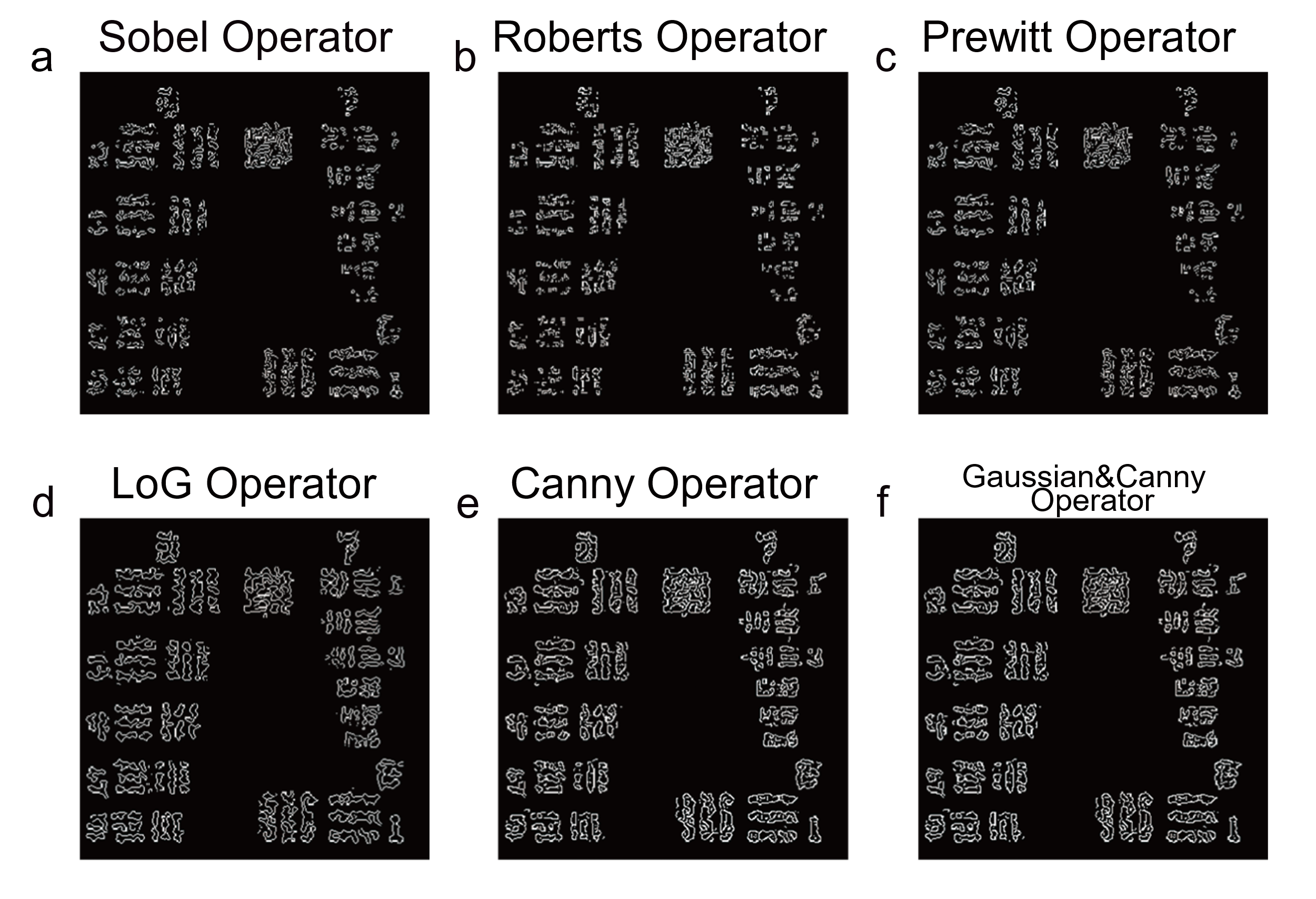


**Fig. S6** Results for different edge detection algorithms of the target in Fig. 5 in the main text when the illumination is a laser without any form of speckle reduction.


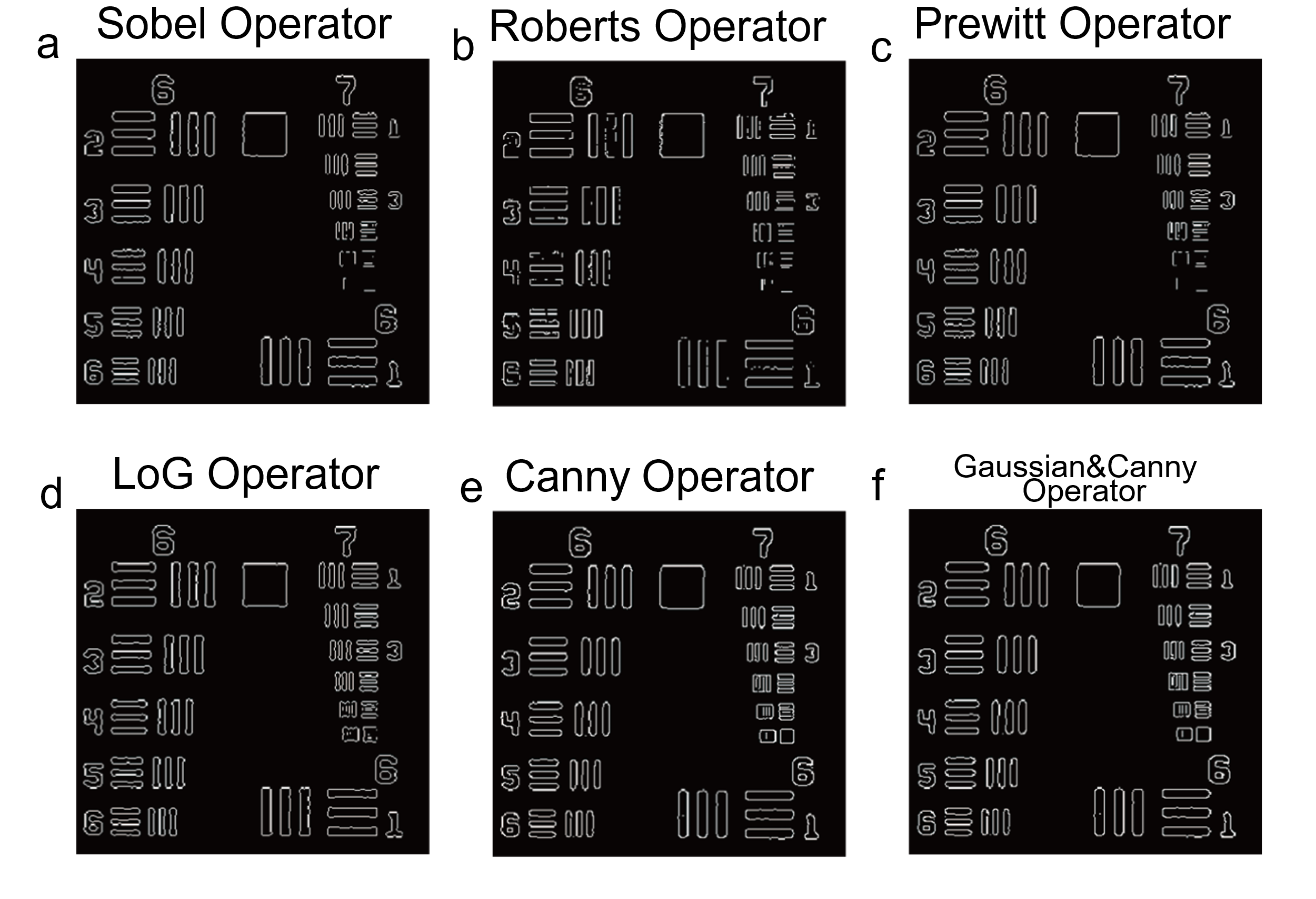


**Fig. S7** Results for different edge detection algorithms of the target in Fig. 5 in the main text when the illumination source is a laser, and the liquid crystal speckle reducer (LC-SR) is inserted and turned on.

**Fig. S6** demonstrates that none of the edge detection algorithms can compensate for the speckle distortion that the laser light introduces in the microscope. Conversely, **Fig. S7** demonstrates that when the LC-SR is inserted the fine details in the target are visible, providing a real-time speckle free image. To select the best suited edge detection algorithm, we compared the thinnest stripes of the targets (group 7, element 6). From Fig. S6, it is apparent that the best algorithm for such a pattern is the Laplacian of Gaussian (LoG) as it not only reveals the outer layer square shape of the stripes but also depicts the inner straight line.

1. **System configurations**

Laser microscope demonstrator

The configuration used to demonstrate the performance of the LC-SR in a laser-based microscope is shown in Fig. 3a in the manuscript. The output from the He-Ne laser was first passed through a variable attenuator (VA) to adjust the intensity so that the CCD camera was not saturated. The LC-SR device was then placed right behind the VA so that the laser light could be directly modulated by the LC-SR. The modulated light then propagated through a positive lens and then expanded to a dimension such that the intensity of the laser light was uniform over the area of interest on the target. The target consisted of a 220-grit ground glass diffuser (GGD) and a Ø1" 1951 USAF Target to mimic a thick, rough sample with fine details. A 10× microscope objective was used to magnify the elements in the target and the enlarged pattern was then collected by another positive lens before being captured by a CCD camera.

Head-up display demonstrator

The setup used to demonstrate the LC-SRs in a HUD application is shown in Fig. 4a in the manuscript. The laser light from a He-Ne laser passed through a variable attenuator (to control the intensity) before it propagated through a ground glass diffuser (GGD) and then the LC speckle reducer. After exiting the LC device, the light propagated through a light pipe (LP) and second GGD before being imaged onto a target mask. The illuminated mask was imaged by a lens and the HUD screen which formed a virtual image 120 mm behind the HUD screen. This image along with background objects was then captured by a CCD placed at the viewing point (VP).

Thin Film Hologram demonstrator

Fig. 5a in the manuscript shows the system employed to demonstrate speckle reduction in a thin-film hologram. The He-Ne laser first passed through a variable ND filter to control the illumination intensity. Following the filter the beam passed through a ground-glass diffuser immediately before the LC-SR. A single lens was used to expand the output beam from the LC-SR to illuminate a large area of the hologram. A CCD camera was then placed at one of two positions (either VP1 or VP2) to capture images of the hologram at two different viewing angles.

Laser projector display

To demonstrate the use of a LC-SR in a projection display the standard illumination system (a bulb in this case) was stripped out of a commercial HITACHI projector and replaced with a laser illumination system. This consisted of a homogenisation system similar to the one in the speckle characterisation system. The first GGD was then followed by the LC-SR mounted on a hot-stage. The light beam then propagated through a light pipe before a second GGD. The output from this illumination system was passed into the projector and used to display full motion video as shown in Supplementary Movie 1.

Mueller matrix polarimetry (vectorial imaging)

Numerous MM imaging polarimeters have already been proposed in previous studies^3,4^. Meanwhile, many MM interpretation methods have been put forward to extract useful polarization parameters to characterize sample information. The widely used decomposition methods^5,6^ – which include Mueller matrix polar decomposition (MMPD) and Mueller matrix transformation (MMT) that we have used in the main article – interpret the MM in terms of different physical parameters such as diattenuation, depolarization, retardance, linear anisotropy level and so on, have been widely used and validated for both forward and backward geometry detections via a series of Monte Carlo simulations and related experiments^5^.

Supplementary Information Fig. S8 shows the MM polarimeters that we adopted in the vectorial imaging experiments in our work, via a dual-rotating wave plate configuration (previously described by *Azzam*, *Goldstein* and *Chipma*^6-10^). The polarizers (P1 and 2) were fixed and oriented along the same direction. The polarizers (P1 and P2) are fixed and oriented in the same direction. Two quarter waveplates (QWP1 and QWP 2) rotate with fixed rotational speeds, such that$\phi_{1}=5\phi_{2}$. The main measurement mechanism is shown in Eq. (1) below, where $S_{\mathrm{in}}$ and $S_{\mathrm{out}}$ are the incident and output Stokes vectors and superscript $q$ represents the $q^{th}$ measurement.

$S_{\mathrm{out}}^{q}=M_{\mathrm{System}}S_{\mathrm{in}}=M_{P2}M_{QWP2}M_{QWP2}^{q}M_{\mathrm{Sample}}M_{QWP1}^{q}M_{P1}S_{\mathrm{in}}$ (1)


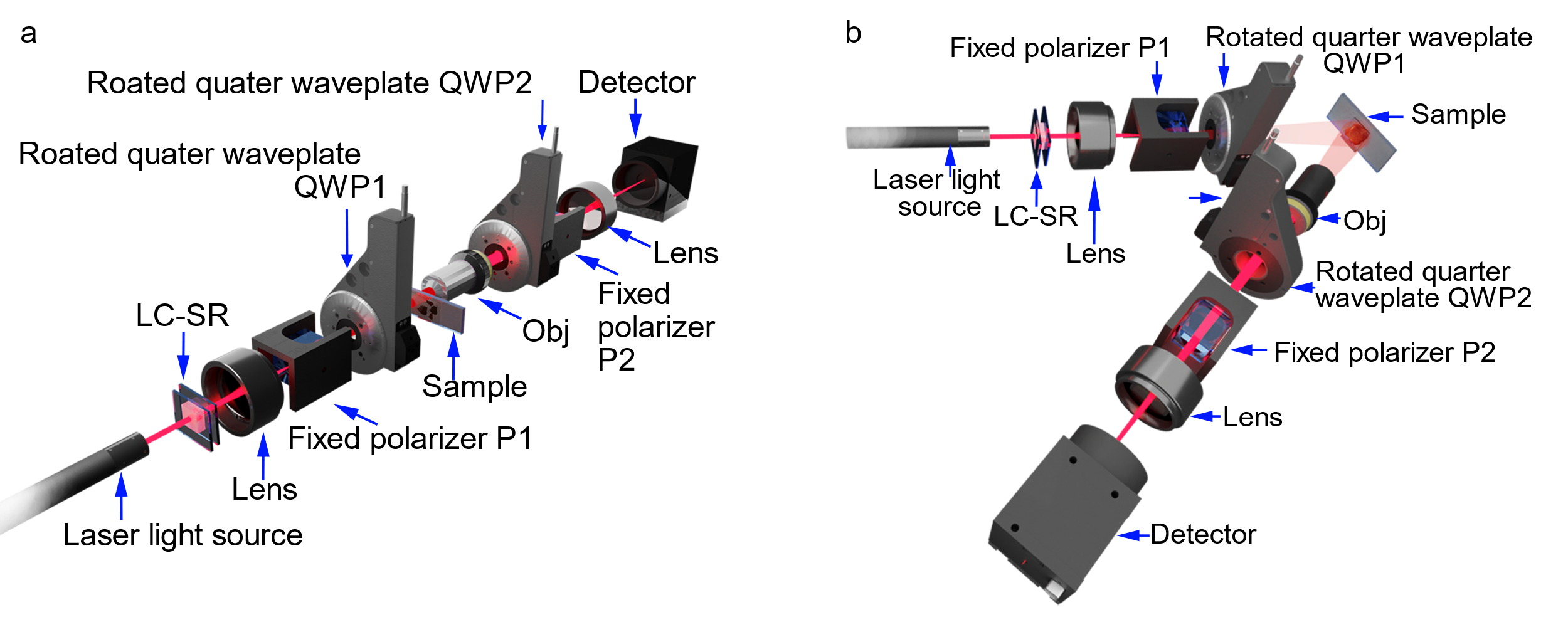


**Fig. S8** Transmissive (a) and back-scattering (b) geometries for MM polarimetry used in this work. (a) The beam comes from the LED (1 W, 633 nm) or He-Ne laser (4 mW, 633 nm) passes through the polarizer (P1, Thorlabs) and quarter wave plate (QW1, Thorlabs). Then the beam from the sample passes through the analyzing quarter-wave plate (QW2, Thorlabs) and polarizer (P2, Thorlabs), and are recorded by a 10-bit CCD camera (Thorlabs). (b) The beam comes from the LED (1 W, 633 nm) or He-Ne laser (4 mW, 633 nm) passes through the polarizer (P1, Thorlabs) and quarter wave plate (QW1, Thorlabs). Then the beam is backscattered from the sample and passes through the analyzing quarter-wave plate (QW2, Thorlabs) and polarizer (P2, Thorlabs), and are recorded by a 10-bit CCD camera (Thorlabs). The LC-SR and the lens are inserted and taken out according to the experimental requirements during the measurement process.

Here,$M_{Sample}$ is the MM of the sample and $M_{P1}$, $M_{P2}$, $M_{QWP1}$ ${\mathrm{and} M}_{QWP2}$ are MM of P1, P2, QWP1 and QWP2. $M_{\mathrm{System}}$ represents the overall MM with respect to the system. As the intensity is equivalent to the first element $S_{0}$ of the Stokes vector, we use$I^{q}={(S_{\mathrm{out}}^{q})}_{0}$, to represent the corresponding intensity of the $q^{th}$ measurement. From Eq. (4) the Fourier series can be acquired, where $a_{n}$ and $b_{n}$ are the Fourier coefficients, and $\phi_{1}^{q}$ is the angle of QWP1 for $q^{th}$ measurement.

$I^{q}={(S_{\mathrm{out}}^{q})}_{0}=a_{0}+\sum_{n=1}^{12} (a_{n}\cos2n\phi_{1}^{q}+b_{n}\sin2n\phi_{1}^{q})$ (2)

From Eq. (2) the Fourier series can be acquired, where $a_{n}$ and $b_{n}$ are the Fourier coefficients, and $\phi_{1}^{q}$ is the angle of QWP1 at $q^{th}$ measurement. For more details see Ref. 4. In the main article, we adopted the MMPD and MMT methods and used the parameters to intuitively illustrate the polarization properties retrieved from the targets. Here we give a brief explanation of the two approaches as well as their related key parameters. More details can be found in Refs 4, 11. MMPD method decomposes the complicated interactions between the sample and polarized light into three basic elements^3^: diattenuation (*D*), retardance (*R*), and depolarization (*Δ*).

The main equation is represented by Eq. (3), where the $M_{\Delta}{, M}_{R}, \mathrm{and}M_{D}$ are the sub-matrices for the depolarization, retardance, and diattenuation. It is worth noting that the MM intrinsically depends on the coordinate systems; however, all decomposed parameters here are not affected by the coordinate system.

$M_{S\mathrm{ample}}=M_{\Delta}M_{R}M_{D}$ (3)

For this work we used the optical retardance and its fast axis orientation. The retardance *R* is reconstructed from the trace of $M_{R}$. The orientation of the optical axis of linear retardance *θ* (with respect to the horizontal axis) ranging from − $\frac{\pi}{2}$ to $\frac{\pi}{2}$ radians can be obtained via Eq. (4). Note the subscripts of the matrix $M_{R}$ represent different components of the sub-matrix.

$R=\cos^{-1} \left[ \frac{\mathrm{tr}\left( M_{R} \right)}{2}-1 \right]$

(4)

$\theta=\frac{1}{2}\tan^{-1}\left[ \frac{M_{R23}-M_{R32}}{M_{R31}-M_{R13}} \right]$

MMT parameters have been proposed to reveal the polarization properties of objects with an emphasis on biomedical or clinical specimens by calculating different combinations of the elements of the MM^3^.

$$t_{1}=\frac{\sqrt{{(m_{22}-m_{33})}^{2}+{(m_{23}+m_{32})}^{2}}}{2}$$

(5)

$x_{3}=\frac{1}{2}\tan^{-1} \frac{m_{42}}{-m_{43}}$

Compared with the MMPD method, the MMT method presents several advantages such as faster calculation speed and more sensitivity to asymmetric vectorial information. It therefore can fit well in the cases such as fast *in* *vivo* clinical diagnosis^11^. Similar to MMPD, there are several extracted angle-free polarization parameters: anisotropy (*t*_1_), and axis orientation (*x*_3_). In the main article, we used the parameters *t*_1_ and *x*_3_ to illustrate the bio-information of the liver tissue. The mathematical presentations of the parameters are given in Eq. (5) above. More details can be found in Ref. 3.

1. **Qualitative Model**

Based on the results from different investigations of speckle reducers and combining the observations with our previous work^12^, we can deduce a preliminary qualitative model. By analysing the data shown in Fig. S1 in the Supplementary Information and considering the discussion in Ref. 12, it can be deduced that *C* is proportional to the pitch (*p*) because *p* relates to the spatial frequency of the refractive index variation. This relationship holds true for pitch values within the range of 224 ≤ *p* ≤ 2050 nm. It has also been observed that the speckle contrast reduces dramatically and nearly linearly with pitch in the range of 500 ≤ *p* ≤ 2000 nm. However, the decrease in speckle contrast becomes more gradual when the pitch ranges from 224 nm to 500 nm^12^. This finding aligns with our results, indicating that the speckle contrast reduces with pitch in the range of 300 ≤ *p* ≤ 500 nm. However, mixtures with very short pitch require larger electric field amplitudes to trigger EHDI and thus speckle reduction. Since a reduction in speckle contrast becomes more gradual with decreasing pitch, coupled with the need for larger electric field amplitudes, we targeted pitch values of *p* ≈ 300 nm for the current study.

Previous work by David Hansford^13^ considered the role of the birefringence (*Δn*) of the nematic LC host on the speckle reduction. For the range 0.15 < *Δn* < 0.2 it was found that the minimum speckle contrast for the chiral nematic LC-SR devices (without ionic dopants), was inversely proportional to the birefringence. However, for devices with a nematic LC host that had a birefringence larger than *Δn* = 0.2, there appeared to be little difference in the speckle reduction for the LC layer thicknesses considered.

Regarding the thickness of the LC layer (*d*), results for the 20 µm-thick and 40 µm-thick LC-SR devices appear to indicate that *C* is inversely proportional to *d*. This relationship is perhaps reasonable when it is considered that devices with a smaller LC layer thickness impose a smaller range of phase perturbations on the light passing through the device. As the thickness of the LC layer approaches dimensions that lead to 2π phase perturbations, further increases in thickness tend to only result in decreased transmission without significant influence on the speckle reduction^14^.

Temperature is another important parameter as it affects the speckle reduction through the viscosity (*γ*). As the temperature rises from room temperature to the clearing point of the mixture, the viscosity decreases, leading to an increase in the number of speckle patterns generated per unit time. To a first approximation, the qualitative phenomenological model appears to suggest that *C* $\propto\frac{p}{\Delta n\cdot d\cdot\gamma}$.

As the electro-optic effect utilized in this work gives rise to bulk fluid flow within the LC-SR device, it is perhaps reasonable to suggest that the alignment along the substrate surfaces would not be the largest contributing factor to the characteristics of the flow. Therefore, the speckle reduction performance is not expected to be as sensitive to the quality or type of alignment layer as it is to other parameters such as concentration of dopant, birefringence, pitch of the chiral nematic helix, temperature, and LC layer thickness. As such, the surface alignment was not investigated in the present work but it could be of topic for future studies for further refinement and optimization of the LC-SR devices.

**References**

1 Zhang Y, Yang X, Zhan Y, Zhang Y, He J, Lv P et al. Electroconvection in Zwitterion-Doped Nematic Liquid Crystals and Application as Smart Windows. *Advanced Optical Materials* 2021; **9.**

2 Goodman JW. *Speckle Phenomena in Optics: Theory and Applications, Second Edition.* 2nd ed. SPIE: Washington, D.C., 2020.

3 He C, He H, Chang J, Chen B, Ma H, Booth MJ. Polarisation optics for biomedical and clinical applications: a review. *Light: Science & Applications* 2021; **10**: 1–20.

4 He C, Chang J, Salter PS, Shen Y, Dai B, Li P et al. Revealing complex optical phenomena through vectorial metrics. *Advanced Photonics* 2022; **4**: 026001.

5 He H, Ma H, Zeng N, Li P, Liao R, Liu X et al. Mueller Matrix Polarimetry—An Emerging New Tool for Characterizing the Microstructural Feature of Complex Biological Specimen. *Journal of Lightwave Technology* 2019; **37**: 2534–2548.

6 Chipman RA, Lu S-Y. Interpretation of Mueller matrices based on polar decomposition. *JOSA A* 1996; **13**: 1106–1113.

7 Azzam RMA. Photopolarimetric measurement of the Mueller matrix by Fourier analysis of a single detected signal. *Opt Lett* 1978; **2**: 148–150.

8 Goldstein DH. Mueller matrix dual-rotating retarder polarimeter. *Applied Optics* 1992; **31**: 6676–6683.

9 Goldstein DH, Chipman RA. Error analysis of a Mueller matrix polarimeter. *JOSA A* 1990; **7**: 693–700.

10 Chipman RA. Depolarization index and the average degree of polarization. *Appl Opt* 2005; **44**: 2490–2495.

11 He C, Shen Y, Forbes A. Towards higher-dimensional structured light. *Light Sci Appl* 2022; **11**: 205.

12 Hansford DJ, Jin Y, Elston SJ, Morris SM. Enhancing laser speckle reduction by decreasing the pitch of a chiral nematic liquid crystal diffuser. *Sci Rep* 2021; **11**: 4818.

13 Hansford DJ. A liquid crystal device for speckle reduction in coherent light. *D.Phil Thesis* 2018..
